# Observations of narrow bipolar events reveal how lightning is initiated in thunderstorms

**DOI:** 10.1038/ncomms10721

**Published:** 2016-02-15

**Authors:** William Rison, Paul R. Krehbiel, Michael G. Stock, Harald E. Edens, Xuan-Min Shao, Ronald J. Thomas, Mark A. Stanley, Yang Zhang

**Affiliations:** 1 Langmuir Laboratory for Atmospheric Research, Geophysical Research Center, New Mexico Institute of Mining and Technology, Socorro, New Mexico 87801, USA; 2 Space and Remote Sensing Group, Los Alamos National Laboratory, Los Alamos, New Mexico 87544, USA; 3 Laboratory of Lightning Physics and Protection Engineering, Chinese Academy of Meteorological Sciences, Beijing 100081, China

## Abstract

A long-standing but fundamental question in lightning studies concerns how lightning is initiated inside storms, given the absence of physical conductors. The issue has revolved around the question of whether the discharges are initiated solely by conventional dielectric breakdown or involve relativistic runaway electron processes. Here we report observations of a relatively unknown type of discharge, called fast positive breakdown, that is the cause of high-power discharges known as narrow bipolar events. The breakdown is found to have a wide range of strengths and is the initiating event of numerous lightning discharges. It appears to be purely dielectric in nature and to consist of a system of positive streamers in a locally intense electric field region. It initiates negative breakdown at the starting location of the streamers, which leads to the ensuing flash. The observations show that many or possibly all lightning flashes are initiated by fast positive breakdown.

## Abstract (web summary)

How lightning is initiated inside storms has been a long-standing and fundamental question. Here, the authors report observations of a previously unrecognized type of discharge, called fast positive breakdown, that is found to initiate many and potentially all lightning discharges in storms.

## Graphical abstract

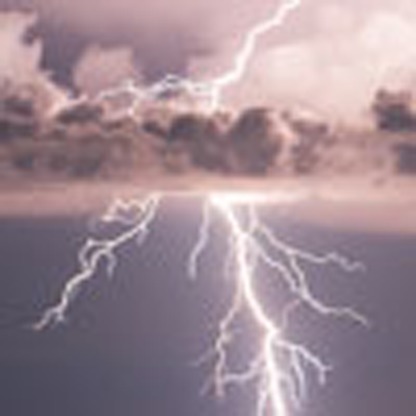

Narrow bipolar events (NBEs) are a highly unusual and poorly understood form of breakdown that typically occurs either as the initiating event of an intracloud (IC) lightning flash or in isolation from other discharges in a storm^1^,^2^. NBEs were first identified in the 1980s and are characterized by short-duration (10–20 μs) bipolar sferic waveforms, accompanied by strong radio frequency (VHF) radiation^3^,^4^. Their fast electric field changes (sferics) are as strong or stronger than those produced by high-current cloud-to-ground (CG) strokes, but are not preceded by detectable breakdown^1^. The early investigations recognized NBEs as being the strongest natural source of terrestrial VHF radiation. The polarity of NBE sferics is typically positive, opposite to that of negative CG strokes and indicative of being an IC discharge between mid-level negative and upper positive charge in storms. Their brief duration implies a relatively short spatial extent of several hundred metres or less and a fast propagation speed (∼10^7^–10^8^ m s^−1^)^1^,^5^,^6^, unheard of for high-current, virgin air events inside storms. Their occurrence has defied explanation for over three decades.

NBEs occur infrequently in storms, but because of the substantial strength of their sferic, they are readily observed out to hundreds of km distance. At such distances, their electric field change is dominated by the radiation component, but the radiation component remains strong even at close distances (≃5–10 km) due to their fast speed and development. NBEs need to be closer than ≃5 km plan distance for the important induction component of their electric field change to be observed, which provides a measure of the breakdown current. Fewer than ≃5–10 such events have been observed scientifically^5^,^7^, in most cases at insufficiently close distances or with sub-optimal instrumentation.

In the following, we report observations of lightning in small storms on 5 and 6 August 2013. The 5 August storm produced three high-power NBEs within 6 km of our well-instrumented mountaintop observatory, Langmuir Laboratory, in central New Mexico. We first show that the NBEs were caused by a newly recognized type of fast positive breakdown, and how the NBEs initiated normal IC discharges in the storm. We then go on to show that the fast positive breakdown occurs with a wide range of VHF strengths and sferic amplitudes and was the initiating event of other discharges in the storms, both ICs and CGs. Finally, we present observations of short-duration, precursor events and a high-altitude screening discharge, showing that they too are due to fast positive breakdown that happens not to develop into full-fledged discharges.

## Results

### Observational data

**Figure 1:**
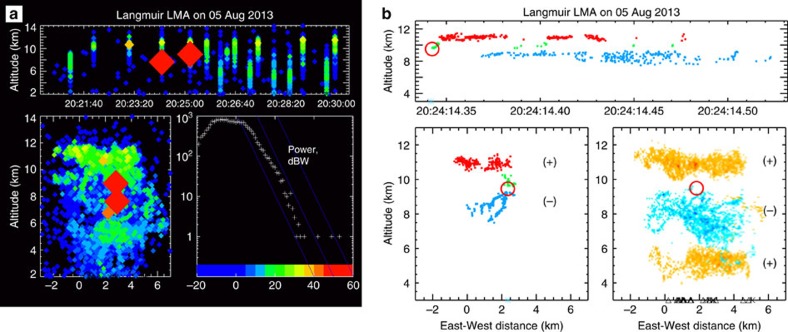
LMA observations of the NBE-producing storm. (**a**) Lightning activity over a 10-min interval as the storm intensified its electrical activity. High- and low-altitude events are IC and CG flashes, respectively. Symbol colour and size indicates VHF source power according to the histogram, and show the occurrence of NBEs 1 and 2 (large red diamonds). (**b**) LMA observations for the bilevel IC flash initiated by NBE1, showing the NBE location (red circles) and lightning—inferred regions of net positive (+) and negative (−) storm charge (lower right panel). Symbols along the abscissae indicate the time and plan location of sferics located by the National Lightning Detection Network (NLDN). NBEs 1 and 2 were detected by the NLDN as positive cloud events having large peak currents of +29.7 and +43.0 kA, respectively.

During 2013, the lightning activity in storms around Langmuir Laboratory was being observed by the three-dimensional Langmuir Lightning Mapping Array (LMA)^2^,^8^ and a high-speed broadband VHF interferometer (INTF)^9^ (see Methods). The INTF also recorded wideband waveforms of a fast electric field sferics sensor, called a fast antenna (FA). The LMA data show that the 5 August storm produced 76 lightning flashes (51 ICs and 25 CGs) over a 1-h time period, typical of the flashing rate in small New Mexico storms. The storm was unusual, however, in that three of the IC flashes were initiated by high-power NBEs. The first two NBEs occurred sequentially as the storm intensified, at 20:24:14 and 20:25:10 UT (Fig. 1a). From the LMA measurements, the NBEs had estimated peak VHF powers of 49.7 and 53.5 dBW (93 and 274 kW), 15–70 dB stronger than the powers of other lightning radiation sources in the storm (histogram of Fig. 1a). From the LMA and INTF data, the NBEs occurred at similar plan locations 5–6 km northeast of the INTF site and were initiated at ≃9.3–9.5-km altitude MSL. NBE3 occurred 18 min later, at 20:43:21, as the first discharge in a new cell at a slightly closer location, 3.3 km north-northwest of the INTF and at ≃9.6-km altitude. Its peak VHF power was 52 dBW (158 kW).

Comparison with the storm's electrical structure inferred from the overall lightning activity^10^ (Fig. 1b) shows the NBEs occurred just above the storm's mid-level main negative charge region^11^. Except for being initiated by high-power NBEs, the resulting IC flashes were no different from other flashes in the storm or in other storms. In particular, the NBE-initiated flashes were ordinary, bilevel discharges between the mid-level main negative and upper positive charge regions of the storm.

**Figure 2:**
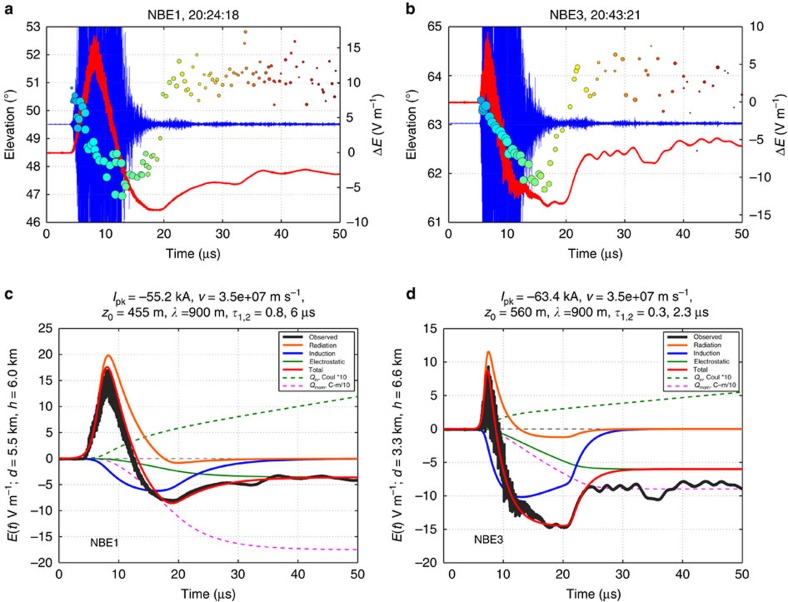
Observational data and simulation results for NBEs 1 and 3. (**a**,**b**) Sferics waveform *E*(*t*) (red) and elevation angle of the VHF radiation sources (dots, sized by log of VHF power and coloured by time) superimposed on the VHF waveform (blue), showing (i) the downward propagation of the radiation sources with time during the NBEs and (ii) the partial field recovery at the end associated with current cessation (see Methods). (**c**,**d**) Simulations of the sferic for each NBE, showing the radiation (orange), induction (blue) and electrostatic (green) components of the total *E*(*t*) simulation (red) (see text). Note attenuated VHF radiation in the measured sferics waveforms (black).

**Figure 3:**
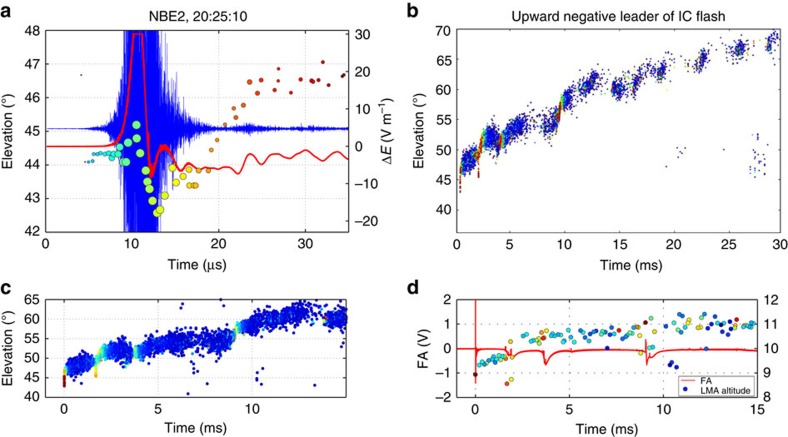
Observations for NBE2. (**a**) Sferic and VHF source elevations, showing the initial elevation increase followed by downward propagation. (**b**) Upward-developing stepped negative breakdown beginning at the top of NBE2, and the delayed onset of detectable radiation at the lower end of the negative breakdown (lower-right faint sources). (**c**) Initial 15 ms of the stepped leader, coloured by VHF source power, showing several episodes of enhanced (lighter coloured) VHF radiation during the steps. (**d**) LMA source altitudes for the upward negative leader (circles), coloured by LMA source power, and fast electric field (red) perturbations associated with the enhanced VHF episodes.

Figure 2a,b shows FA and INTF observations for NBEs 1 and 3. Observations for NBE2 are shown in Fig. 3a. The VHF radiation sources for NBEs 1 and 3 rapidly decreased in elevation angle with time, while the sources for NBE2 initially increased in elevation before rapidly decreasing. For NBEs 1 and 2, the breakdown occurred at constant azimuth, indicating the breakdown was vertically oriented. This is supported by the LMA data, which showed that the ensuing IC discharges developed vertically upward in the storm immediately following the NBEs (Fig. 1b). For NBE3, the breakdown was slightly tilted from vertical and exhibited a noticeable spread in azimuthal values.

From the elevation changes and LMA-determined plan distances, the VHF radiation for each of the NBEs descended ≃500 m in the storm. For NBEs 1 and 3, this occurred over time intervals of 12 and 10 μs, respectively, corresponding to propagation speeds of ≃4 × 10^7^ and 5 × 10^7^ m s^−1^. Both the polarity of the initial radiation peak and the sferic's final electrostatic offset show unambiguously that the downward propagation was produced by positive breakdown that lowered positive charge in the storm. This is consistent with the NBEs occurring above the main negative storm charge (Fig. 1b). NBE2 was slightly different, in that its VHF sources initially increased in elevation, and is discussed in a subsequent section.

Time series data for NBEs 1 and 3 (Supplementary Figs 1 and 2) show that their VHF power increased and decreased exponentially with time, with rise time constants as short as 0.3 μs and fall time constants of several microseconds. In both cases, the observations showed no evidence of activity before the NBE onsets, down to the ambient noise levels in both the INTF and FA data, ≃66 dB below their peak amplitudes. Rather, the breakdown began abruptly, within a microsecond or less, and simultaneously on the two instruments.

### Sferic simulations

We evaluated the FA waveforms for NBEs 1 and 3 by assuming that their *E*(*t*) sferics were produced by a downward-propagating current pulse having a double-exponential waveform (Methods; Supplementary Fig. 3). Figure 2c,d shows the radiation, induction and electrostatic contributions that provide the best overall fits to the observed sferics. In both cases, the electric field changes had (i) a strongly positive initial radiation component with relatively small undershoot (orange curve), (ii) a relatively strong negative induction component (blue curve) that caused most of the undershoot in the total electric field change and (iii) a steadily negative-going electrostatic component that produced the negative offset at the end of the sferic (green curve). The induction and electrostatic components were negative due to the NBEs being at close range, and therefore within the distance beyond which dipolar field changes undergo a polarity reversal^12^. Within the reversal distance, the induction and electrostatic changes were dominated by the descent of positive charge overhead, driving the electric field at the ground negative. The sum of the three components constituted the simulated sferic (red waveforms).

Overall, the sferic simulations agreed well with the measured electric field changes (black waveforms). The simulation-estimated propagation speeds, and rise and fall times were in good agreement with the values inferred from the VHF data, as were the overall extents *z*_0_ of the breakdown, discussed further below. The simulations provide estimates of the peak current *I*_pk_, the amount of charge *Q*_in_ involved in the breakdown and the corresponding charge moment change *Q*_mom_. For NBE1, the model-estimated results correspond to *I*_pk_=−55 kA, *τ*_1_, *τ*_2_=0.8 and 6.0 μs, respectively, *v*=3.5 × 10^7^ m s^−1^, *z*_0_=455 m, *Q*_in_≃1.0 C and *Q*_mom_≃−180 C-m. The current pulse for NBE3 had faster rise and fall times and shorter apparent spatial extent (Supplementary Fig. 3) to fit its narrower radiation sferic, but was otherwise similar to NBE1. Its fit corresponded to *I*_pk_=−63 kA, *τ*_1_, *τ*_2_=0.3 and 2.3 μs, respectively, *v*=3.5 × 10^7^ m s^−1^, *z*_0_=560 m, *Q*_in_≃0.5 C and *Q*_mom_≃−90 C-m. In both cases, the current appeared to be only slightly attenuated with distance along the developing path (*λ*≈900 m). Whereas the electrostatic offset at the end of NBE1 was well fitted by the simulation, the offset of NBE3 could not be so fitted. This may have resulted from the downward breakdown being directed slightly away from vertical towards the INTF site, giving a larger electrostatic change than for vertical development.

It is important to note that the sferics of NBEs 1 and 3 would be similarly well fitted if they were caused by upward-propagating negative breakdown rather than downward positive^6^. This is because both constitute a downward-directed current and because the breakdown extends over a relatively short distance within the storm. Distinguishing between the two possibilities is important, since runaway electron processes would produce upward negative avalanching. The INTF observations fundamentally resolve this ambiguity by showing that the breakdown developed downward rather than upward.

Although the peak currents are extraordinarily large, the amount of charge transferred is relatively small (∼0.5–1.0 C). The currents are large due to the high speed of the breakdown, and the charge transfer is small due to its short duration. Adding reflections of different polarities and strengths at the bottom end of the breakdown produced radiation transients that were clearly inconsistent with the observed sferics. These results disagree with the finding that NBEs are a ‘bouncing wave' phenomenon^13^. Because reflections imply the presence of a conducting channel and would necessarily occur as the current ceases, their absence indicates the NBE breakdown does not produce a conducting channel. Instead of being or becoming a positive leader, which would inevitably become conducting as a result of the NBE's high current, the breakdown appears to be produced by a spatially and temporally distributed system of positive streamers, in which the total current is spread over some cross-sectional area as a volume current density. The basic process by which such breakdown would occur was studied by Phelps and Griffiths^14^,^15^, who showed that repeated positive streamers would become self-intensifying in strong electric fields (see Discussion).

It initially appeared that the INTF data itself might indicate a reflection, in that the radiation sources were displaced upward in elevation with time as the radiation amplitude decreased (Fig. 2a,b). Instead, the upward displacement resulted from the positive breakdown apparently being produced by a succession of cascading current events, in which the later events did not propagate as far down as the earlier ones. This is particularly noticeable for NBE1, whose current pulse was comparable in extent to the overall length of the breakdown (Supplementary Fig. 3), and whose later VHF radiation sources occurred at two levels, with the mid-level activity persisting longer than the lower activity (Fig. 2a). By comparison, NBE3 had a narrower current pulse and a correspondingly monotonic elevation descent. For both NBEs, the final upward displacement was an apparent effect resulting from the decreasing relative strength of the VHF radiation from the positive breakdown compared with that of negative breakdown at the top of the NBEs. Such fast apparent motions are commonly seen in the INTF observations and result from the INTF locating the radiation centroid; here they occurred as the NBEs died out, culminating in step elevation changes at the time of inferred current cessation (see Methods).

### Flash initiation

NBE2 occurred 56 s after NBE1, as the next flash of the storm (Supplementary Fig. 4). It differed from its predecessor in that the breakdown began gradually rather than abruptly, with weak radiation that required several μs to intensify (Fig. 3a; Supplementary Fig. 5). As the breakdown strengthened, it increased in elevation angle before rapidly descending. Its fast electric field change was also more complex, having a noticeable secondary peak following the elevation decrease. That the radiation began weakly indicates the local electric field was not strong enough initially to generate extensive downward breakdown. Rather, the initial activity itself appeared to self-intensify the electric field to the point that the downward NBE was launched. Assuming the VHF radiation was produced by positive breakdown, as in NBEs 1 and 3, NBE2's initial elevation increase would have resulted from the breakdown being initiated at successively higher altitude until becoming strong enough to produce the fast downward NBE. In terms of the Phelps and Griffiths positive streamer mechanism, the intensification would have been caused by negative charge being funneled back to the origins of the positive streamers, creating a positive-feedback process that eventually produced the fast downward breakdown.

That the initial radiation of NBE2 was due to positive breakdown is indicated by several apparent attempts at downward development during the intensification stage, seen as lower elevation sources before 10 μs in Fig. 3a. Once fully initiated, the downward breakdown descended at a high speed, ≃10^8^ m s^−1^. The associated sferic was correspondingly stronger and saturated the digitiser. In addition, the sferic had a noticeable secondary peak. Results of simulating the sferic are presented in Supplementary Fig. 5. The model-estimated parameters for the main peak were *I*_pk_=−57 kA, *τ*_1_, *τ*_2_=0.5 and 0.6 μs, respectively, *v*=9 × 10^7^ m s^−1^, *z*_0_=630 m, *Q*_in_≃0.5–0.7 C and *Q*_mom_≃−80 C-m, faster and shorter in duration than NBEs 1 and 3. The secondary peak is indicative of a resurgent current event and was included in the simulation by adding a second current similar to the first except having a peak current of 21 kA, delayed in time by 3–4 μs. From the INTF observations, radiation from the resurgent activity appeared partway along the downward path, as indicated by the radiation centroid shifting upward to an intermediate elevation during the secondary peak and continuing there as the NBE died out.

Observations of how the ensuing IC flash developed are instructive. In particular, the NBE initiated a sequence of stepped events that constituted the upward negative breakdown of the flash (Fig. 3b). The negative breakdown was well detected by the LMA (Fig. 3d), with the stepping being associated with successive episodes of enhanced VHF and electric field perturbations in the INTF and FA data (Fig. 3c,b). Such episodes are typical of negative breakdown at the beginning of IC (and CG) flashes^16^,^17^, and are called initial breakdown pulses (IBPs)^18^,^19^. Instead of being a singular pulse, however, IBPs consist of a sequence of fast (≃1-μs duration) sub-pulses embedded in the slow component of the IBP (Supplementary Fig. 6). Rather than flashes being initiated by the IBP, as suggested by its name, the initiation was caused by the fast positive breakdown of the NBE, in this case ≃1.5 ms earlier.

The manner in which the conversion to a conducting negative leader occurred is not clear, and is the subject of separate investigations (for example, ref. 20). Here we note that the VHF activity immediately following NBE2 and the other two NBEs were scattered to varying degrees above the parent NBE (Supplementary Figs 7, 8 and 9). The scattered sources correspond to the beginning of the initial E-change interval of the studies by Marshall *et al.*^19^,^21^. The present observations indicate the negative leader had not yet formed nor had it appeared to form through the full 1.5 ms duration of NBE2's initial E-change (Supplementary Fig. 6). By the end of the first IBP, only 2.5 ms into the flash, the negative breakdown extended into the storm's upper positive charge region and began developing horizontally through the region (Supplementary Fig. 4).

We note that a second NBE occurred during the initial step. The NBE is seen in the elevation data of Fig. 3c partway into the first VHF enhancement interval, and in more detail in Supplementary Fig. 6. The NBE propagated vertically downward in the storm, separated in azimuth by about 3–4° (≃300–400 m) from NBE2, and ≃500 m higher in altitude. It appeared to be triggered by the negative breakdown leading up to its occurrence and likely assisted in further development of the step. The NBE was detected by the LMA as having a peak power of 20.5 dBW, 33 dB below that of NBE2 itself. The NBE's duration (35 μs) and extent (≃500 m) were otherwise similar to that of the high-power NBEs.

Further activity in the initiation region of the flash was not detected until 20–25 ms into the discharge, and then only in the initial negative breakdown region immediately above NBE2 (faint INTF sources in Fig. 3b), rather than below the NBE. The corresponding LMA activity was similarly delayed in time (Supplementary Fig. 4). Such delays are a common feature of VHF observations of IC flashes (for example, Fig. 1b and refs 9, 16), and are due to the post-initiation positive leaders being inherently weak and/or masked by the stronger radiation of the negative breakdown. This, and the fact that positive leaders are relatively slow (typically a few times 10^4^ or 10^5^ m s^−1^), contrasts sharply with the strongly radiating fast positive breakdown of this study and of our earlier studies (see Discussion)^22^. The present observations suggest the positive leaders result from some of the downward fast breakdown being converted to slow leaders as the NBE dies out.

Finally, we note that Fig. 3a shows oscillatory ‘ripples' in the sferic following NBE2. Similar ripples are seen in the sferics of NBEs 1 and 3. The ripples occur as the sferic settles out to the electrostatic offset and last for several tens of μs following the NBEs. Faster oscillatory behaviour during the NBE itself has been reported by Nag and Rakov^13^ and formed the basis for their ‘bouncing wave' model of NBEs. However, the model simulations of their study showed oscillations only at far-field (200 km) distances and not at close distances (2 km). In addition, Eack's^5^ close NBE observation could be simulated only by assuming no reflections. The ripples arise from some other process, having to do for example with the onset of negative breakdown at the top of the NBE, and/or with residual conductivity in the NBE's wake. It would be highly improbable for NBEs to produce a conducting channel carrying tens of kA of current and for the channel to die out before the remainder of the discharge, even in a less-strong ambient field region.

### Initiation of other storm flashes

**Figure 4:**
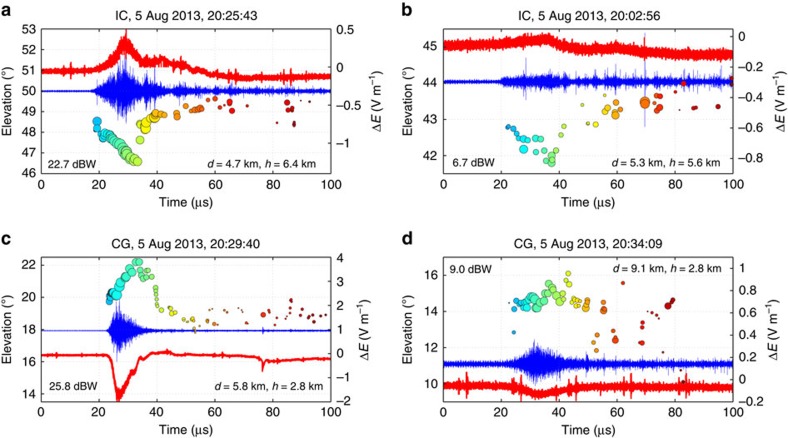
Less-strong NBE events that initiated other discharges. (**a**,**b**) Fast positive downward NBEs that initiated IC flashes. (**c**,**d**) Same, except for two negative CG flashes initiated by upward rather than downward positive breakdown. The dBW values are VHF source powers of the LMA event located for each NBE. CG event (**d**) occurred at a greater plan distance *d* and therefore at a correspondingly lower and less-accurately determined elevation angle. *h* is the initiation height above the plane of the INTF, situated at 3.2-km altitude MSL.

Seeing how the high-power NBEs initiated IC discharges led us to investigate the beginnings of other flashes in the storm. Surprisingly, six other discharges were quickly found to be initiated by weaker versions of fast positive breakdown. INTF and FA data at the beginning of two IC flashes (Fig. 4a,b) show the same downward development characteristic of the fast breakdown, but accompanied by weaker VHF radiation and positive sferics. Even more surprising, two negative CG discharges were found to be initiated by upward positive breakdown that also radiated less strongly at VHF and produced smaller amplitude negative sferics (Fig. 4c,d). The CG observations are remarkable, as they show the discharges were initiated in the same manner as IC flashes, except by upward rather than downward breakdown. This agrees with the CG discharges being initiated below the storm's negative charge region (Fig. 1b)^11^,^21^,^23^.

The IC and CG discharges of Fig. 4 exhibited a range of VHF source powers, being ≃20–25 dBW for the relatively stronger events and ≃5–10 dBW for the less-strong events, 25–45 dB less than the ≃50 dBW powers of the strong NBEs. The sferics were also substantially weaker and more monopolar in nature. Despite these differences, the apparent extents, durations and propagation speeds of the positive breakdown differed only slightly from those of the high-power events. From the INTF and LMA data, the estimated vertical extents for the medium-strength IC- and CG-positive breakdown were 470 and 310 m, respectively, and the durations were 13 and 10 μs, corresponding to estimated speeds of 3.6 and 3.1 × 10^7^ m s^−1^. For the weaker cases, the positive breakdown was ≃200–170 m in extent and 15 and 14 μs in duration, corresponding to speeds of 1.3 and 1.2 × 10^7^ m s^−1^.

**Figure 5:**
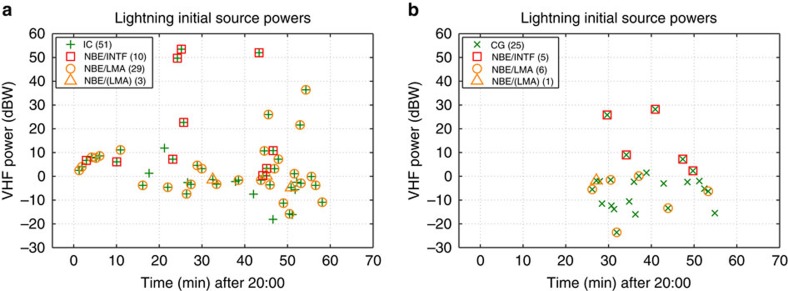
Initial LMA source powers for all discharges in the 5 August NBE storm. (**a**) Initial peak power versus time for IC flashes, showing which were initiated by NBE breakdown based on (i) INTF and LMA data (red squares) and (ii) primarily on LMA data (orange circles and triangles). (**b**) Same, but for CG flashes. Unmarked discharges are also likely to have been initiated by NBE breakdown, as per the metric analyses of Supplementary Fig. 13 for the 5 August NBE storm and Fig. 7 for the 6 August storm. Also see Supplementary Fig. 10 for additional plots of the 5 August storm flashes.

To investigate how the storm's remaining discharges were initiated, we examined the LMA and INTF data at the beginning of each of the storm's flashes (Fig. 5; Supplementary Fig. 10). Of 76 total discharges, 15 flashes (10 ICs and 5 CGs) produced detectable fast elevation changes and/or sferics showing they were initiated by fast positive breakdown (red squares). An additional 35 flashes (29 ICs and 6 CGs) were identified as being fast positive initiated based on their initial LMA sequences and, when available, their INTF data as well (orange circles). Most of the remaining flashes were either too weak or intensified too quickly to allow their initiation to be determined.

The above results, and those that follow, show that fast positive breakdown occurs with a wide range of VHF strengths and sferic amplitudes and initiate many and possibly all lightning discharges in storms. This result is supported by recent observations of lightning initiation by Marshall *et al.*^19^, who used measurements of close CG and IC flashes to show the flashes were initiated by unknown, weak impulsive events, which they termed ionizing events. Their example sequences of activity subsequent to initiation were the same as shown in Fig. 3d, except the ionization events were weak. Their study also carefully documented that no electric field variations occurred before the ionization events, in agreement with and complementing the observations and conclusions of the present study.

### Short-duration discharges

To further assess the role of fast positive breakdown in initiating lightning, we investigated short-duration discharges that are commonly and increasingly observed in VHF observations of storms^24^,^25^,^26^. Such events are indicative of attempted breakdown that does not develop into full-fledged lightning. As such, they are of obvious interest to the question of lightning initiation, but little has been known about them or how they are produced^24^,^27^.

A number of short-duration discharges were observed by the LMA in nearby storms on 6 August. No high-power NBEs occurred in the storms. The discharges occurred at isolated times and locations between regular lightning, at the same altitudes as IC flashes (Supplementary Fig. 11). We loosely refer to them as precursors (PCs), as they sometimes occur seconds before an IC discharge initiates at the same location^28^. Some of the short-duration discharges were captured during the several second-long preflash intervals of the high-speed INTF measurements (see Methods).

**Figure 6:**
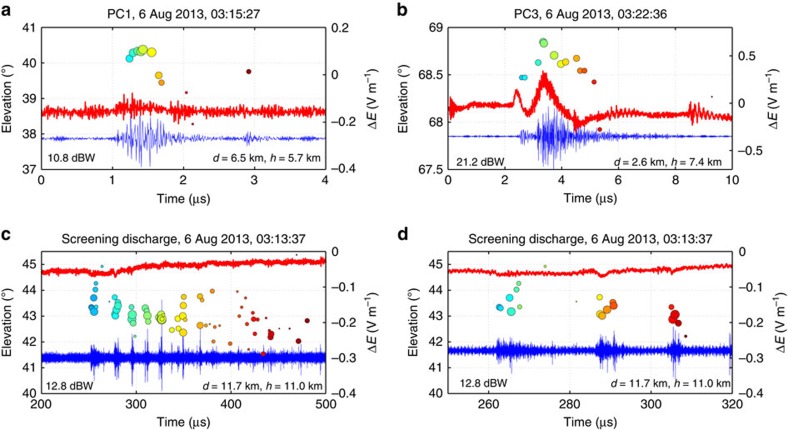
Initial activity of precursor and screening discharges. Same as Fig. 4, except showing the breakdown at the beginning of isolated precursor events PC1 and PC3 in the 6 August storms (**a**,**b**; Supplementary Fig. 11), and at the beginning of a high-altitude screening discharge (**c**,**d**; Supplementary Fig. 12). The PCs were initiated at 8.9- and 10.6-km altitude with initial VHF source powers of 10.8 and 21.2 dBW, and radiation durations of ≃0.7 and 2.0 μs, respectively. Both were unsuccessful IC discharges lasting 250 μs and 3 ms. (The initial small pulse at the beginning of PC3 was horizontally separated from the main activity of the precursor itself. Also, the sources between 39° and 40° elevation at the end of PC1 were superluminally displaced ≃200 m below PC1 and were also due to separate activity.) The screening discharge was initiated at 14.2-km altitude, above the upper positive storm charge. It developed in a different manner from the lower-altitude IC precursors, namely as a succession of discrete upward positive streamer events over a ≃100-μs time interval (see text).

Figure 6a,b shows INTF observations of two precursor events, labelled PC1 and PC3. PC1 lasted only 250 μs, while PC3 lasted 3 ms. Both were initiated by intermediate-strength NBE events, having initial source powers of 10.8 and 21.6 dBW, respectively, each 20–30 dB stronger than the next LMA source 100–200 μs later. The initial breakdown of PC3 lasted ≃2 μs, sufficiently long for the INTF sources to show downward fast positive propagation. The VHF sources descended ≃160 m in 2 μs, corresponding to a speed of 8 × 10^7^ m s^−1^.

PC1 was more distant than PC3 (6.5 km versus 2.6 km plan distance) and also weaker, producing a barely detectable sferic. Its radiation lasted <1 μs and was not resolved by the INTF. That PC1 was caused by fast positive breakdown is indicated by its LMA-detected source power being 20 dB stronger than the subsequent LMA sources of the discharge (+10.8 dBW versus −10 to −16 dBW for the next three LMA sources over the remaining 250 μs; see Metric analyses section). With its VHF radiation lasting ≃0.7 μs, positive breakdown propagating at 3 × 10^7^ m s^−1^ would travel only 21 m, less than the angular resolution of the INTF. We later infer that such short-duration and spatially compact discharges are also able to initiate IC discharges, attesting to the efficacy of fast positive breakdown as an initiation process.

In addition to precursors, the 6 August storms also produced several high-altitude ‘screening' discharges—namely, discharges above the storm's upper positive charge, between it and negative screening charge attracted to the cloud top^23^,^29^. The positive breakdown of such discharges sometimes escapes above the cloud top as a blue jet or starter^30^. Figure 6c,d shows observations for a 6-ms duration screening discharge that initiated at 14.2-km altitude. Like precursors, the observations show that the discharge was initiated by fast positive breakdown, except upward propagating rather than downward. However, the breakdown had a significantly different character in that it consisted of a succession of temporally separated fast positive events, in this case at intervals of ∼20 μs over the first 100 μs of the discharge. Expanded plots (Supplementary Fig. 12) show that the first event developed ≃360 m vertically upward in 5 μs, corresponding to a speed of 7 × 10^7^ m s^−1^. The VHF radiation decreased in intensity as the breakdown ascended. A final, weak VHF source indicates the breakdown developed an additional few hundred metres upward in the next 5 μs. The second positive event ascended at a similar speed (6 × 10^7^ m s^−1^), but over a shorter distance (≃200 m) and lesser overall time (3–4 μs). The third and subsequent events were briefer and generally of decreasing vertical extent, but somewhat more energetic VHF wise. For each event, the associated sferic was exceedingly weak, <0.05 V m^−1^.

What is most striking and significant about the screening discharge (and a second one) is first that the upward positive events are separated out time wise, rather than overlapping in time, as inferred for the high-power NBE observations. Second, the upward breakdown events begin at successively lower altitude, that is, they develop retrogressively downward. The same development happened at the beginning of NBE2, except that the successive events overlapped in time and increased rather than decreased in elevation. Being recently obtained and wholly unexpected, the screening observations support the inference that mid-altitude fast positive breakdown is produced by a succession of overlapping downward breakdown events that can start at retrogressively higher altitudes.

### Metric analyses

To determine how other precursor events were initiated, and to compare their initiation with that of IC and CG flashes, we analysed LMA data for the complete sequence of lightning activity (ICs, PCs and CGs) during an arbitrary 3.5-min time interval in the 6 August storms. During this time, several scattered storms over the LMA network produced a total of 69 discharges: 30 ICs, 25 PCs and 14 negative CGs/low-altitude ICs. An analysis of LMA data for the first millisecond or so of the discharges was conducted utilizing a codified metric-based assessment of the LMA criteria developed and tested for the 5 August NBE storm flashes (see Methods).

**Figure 7:**
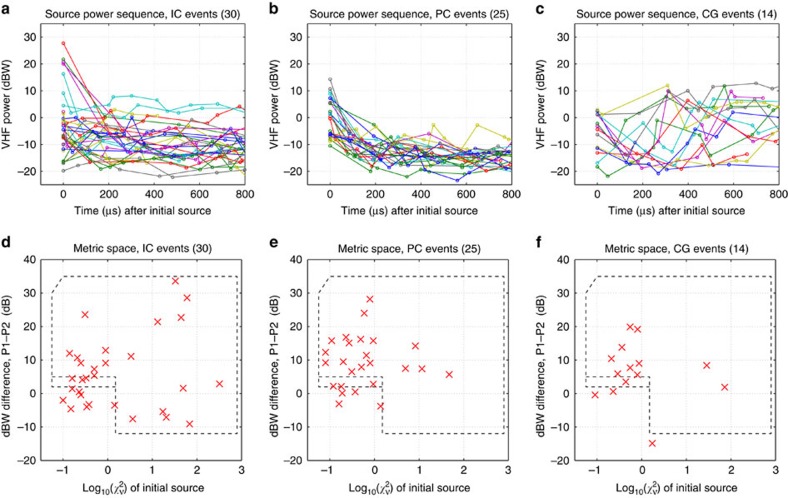
Detailed analysis of LMA data for 6 August storms. (**a**–**c**) Initial VHF source power sequences for the first 800 μs of the IC, precursor (PC) and CG discharges during an arbitrary 3.5-min interval beginning at 03:10 UTC on 6 August. (**d**–**f**) Same, except showing results in the metric space of the power difference (P1−P2) between the first and second source versus the first-source *χ*_ν_^2^ goodness of fit value. The initiation event occurs at *t*=0 in the sequence plots. The large dashed box in each metric panel encompasses clear NBE initiation events and the adjacent, narrow rectangular box encompasses likely initiation events.

Results of the analyses are summarized in Fig. 7. IC flashes were found to have a wide range of initial source powers (from ≃+30 dBW down to −20 dBW, Fig. 7a), similar to the range of values observed for most of the IC flashes in the NBE storm (Fig. 5a). The precursor discharges exhibited a similar but somewhat more confined range of initial powers (Fig. 7b). More significantly, the precursors were not any weaker than their IC counterparts. This and the observational data in general show that ICs and PCs are fundamentally the same phenomena, except that ICs happen to develop into full-fledged discharges, while PCs do not. The difference is seen in the strength of the subsequent radiation sources, being somewhat stronger and more variable power wise for events that develop into ICs, and less variable and decaying for those that do not. Because the PCs do not have the complication of intensifying they provide a clearer indicator of how both PCs and ICs are initiated.

In terms of the metric space, 18 of 25 precursor events (Fig. 7e) had noticeably stronger initial source powers relative to the second source, by 5 dB or more, and/or high *χ*^2^_*ν*_ values (large dashed area), and almost certainly were initiated by fast positive NBE-type breakdown. An additional four PCs had initial source power differences >2 dB (small adjacent dashed box), a likely indication of NBE breakdown. The remaining events had small or negative power differences and are undetermined. The IC flash metrics (Fig. 7d) gave similar results, except for being somewhat more variable power difference wise. It is important to note that small or negative Δ*P* values are easily caused by rapid flash intensification, particularly if the second LMA source was not detected until 100–200 μs or so after initiation. This is particularly true for CG discharges, whose lower altitude and higher pressure somehow disposes them to develop more rapidly and powerfully (Fig. 7c,f; Supplementary Fig. 13). That the initial pulse amplitude of NBEs is usually larger than that of the second pulse was also found to be a characteristic feature of NBE-initiated flashes in the study by Wu *et al.*^31^

Finally, we note that high-power NBEs occur which, like precursors, are temporally isolated and do not initiate IC discharges^1^,^13^,^31^. These have been termed compact intracloud discharges, or CIDs. Fundamentally, CIDs are just the high-power tail of the spectrum of precursor events.

## Discussion

This study identifies high-power NBEs as being caused by fast positive breakdown. The breakdown does not appear to be preceded by other activity, but to occur in virgin air. Its limited spatial extent (less than or equal to ≃500 m) and fast speed (typically 3–7 × 10^7^ m s^−1^) indicate the breakdown occurs in localized regions of intense electric field. The breakdown does not produce a conducting channel, but instead appears to consist of a volumetrically distributed system of positive streamers or streamer-like activity^14^,^15^. The streamers would be initiated by corona from ice crystals or liquid hydrometeors, as described for example by Petersen *et al.*^32^, and die out after propagating through the strong field region.

On the basis of the overall results, we tentatively conclude that all in-cloud lightning discharges are initiated by NBE-type fast positive breakdown. Additional studies will be required to test this conclusion, particularly in the case of lower-altitude discharges such as CG flashes. While it has long been known that CG and IC discharges begin differently^33^,^16^, the present results indicate their initiation process is the same, with the main difference being that CG discharges intensify more rapidly, and may be more difficult to initiate.

Although unexpectedly arrived at via the study of high-power NBEs, the findings support the idea that positive streamers are responsible for initiating lightning. This idea was first proposed in the 1960s by Loeb^34^ and by Dawson and Winn^35^, based on the fact that positive corona and streamers are initiated in less-strong electric fields than their negative counterparts. The basic mechanism for how this would happen was developed during the 1970s by Phelps and Griffiths^14^,^15^, who showed that repeated hydrometeor-initiated positive streamers would cause increasing amounts of negative charge to be funneled back towards the streamer origins, causing additional streamers to be generated and the discharge to become self-intensifying.

The present results are consistent with the positive streamer scenario in a number of ways. In addition to the streamers increasing in number and intensity, their origins would likely spread laterally within the high-field region as the discharge develops, distributing the current over a relatively large cross-sectional area and allowing large currents to be produced by the smaller volume current densities of a large number of individual streamers. An example of lateral spreading is seen in the azimuthal data for the INTF sources of NBE3 (Supplementary Fig. 9). The positive streamer process would also produce a succession of cascading events. Evidence of such cascading is seen in the VHF source data, both in the occurrence of attempted downward events and in the multi-altitude nature of the VHF sources during NBEs 1 and 2 (Figs 2a and 3a). It is also evidenced by the relatively large spatial extent of the current waveforms required to simulate the sferics (Supplementary Fig. 3). The Phelps and Griffiths^14^ mechanism would also predict the positive breakdown to initiate retrogressively in altitude. This is seen in the upward development at the beginning of NBE2 (Fig. 3a; Supplementary Fig. 8) and more explicitly in the retrograde downward development of the temporally resolved breakdown at the beginning of the screening discharge (Fig. 6c,d; Supplementary Fig. 12).

Unlike runaway electron avalanches, which require relatively large extents (hectametres to kilometres) to fully develop, positive streamers reach their maximum intensity within a few metres^15^, allowing streamer ‘avalanches' to develop within tens of metres or 1ess^32^. Thus, the positive breakdown could occur within relatively small volumes of strong electric fields, which would account for the large range of initial strengths. Even a small number (<≃10) of repeated individual streamers over such distances is predicted to intensify the field at their starting origin by an order of magnitude^15^,^32^, explaining how even initially weak breakdown could intensify and lead directly to a more complete discharge. That high-power NBEs are produced by a system of optically dim streamers also explains the deficiency of their optical radiation^36^.

Reproducing the radiation component of high-power NBEs requires that the current reaches its peak intensity within a few microseconds, with submicrosecond e-folding times (between 0.3 and 0.8 μs for the three high-power NBEs of this study). For propagation speeds of 3–4 × 10^7^ m s^−1^, the positive breakdown would travel 30–40 m in 1 μs. Thus, the feedback process giving rise to exponential growth would have to occur over distances of a few tens of metres or less. That a feedback process is involved is indicated by the relatively gradual start of NBE2 and of the less-strong breakdown events of Fig. 4. The ensuing growth is rapid due to the high speed of the breakdown, which in turn results from the electric field needing to be strong to initiate the breakdown and for the feedback threshold to be crossed.

That fast positive breakdown occurs was shown in an earlier study of CG discharges with our original analogue INTF^22^. In particular, fast, VHF-bright-positive streamers were documented immediately following negative CG strokes. The return strokes induce positive charge and ground potential along their channels inside the storm's negative charge region, producing locally intense electric fields. The breakdown propagated at speeds of 1–6 × 10^7^ m s^−1^ away from the return stroke channels, along previously untravelled paths. Strong positive bursts also occurred during the development of negative leaders of CG and IC flashes. Ongoing studies with the present INTF show numerous examples of these kinds of fast positive breakdown^37^,^38^, which often produce the strongest VHF radiation of flashes. The flash-initiating NBEs of the present study appear to be the same type of breakdown, with the significant difference that it occurs in the absence of preceding activity.

An important aspect of the observations is that the breakdown speed does not vary with altitude. The speed is essentially the same for IC discharges initiating at ≃8–10-km altitude, CG discharges at 5–7-km altitude (both in the present study and in the earlier study above), and finally with the screening discharge at 14–15-km altitude. The speeds are typically ≃3–7 × 10^7^ m s^−1^, and range from ≃10^7^ to 10^8^ m s^−1^. Sprites also initiate with fast positive breakdown, typically with speeds of ≃10^7^ m s^−1^ at altitudes of ≃70–80 km (for example, refs 39, 40, 41). Such altitude invariance is due to the discharge processes obeying similarity laws related to air density^42^,^43^,^44^. In particular, the increase in mean free path travelled by free electrons is cancelled by the decrease of the breakdown field with increasing altitude, causing the electron drift velocity to be independent of altitude at breakdown conditions. The study by Briels *et al.*^42^ showed that similarity holds remarkably well for the diameters of positive streamer heads, over the full range of their experimental pressure values (1.0 bar down to 13 mbar (≃30-km altitude)). The present observations show that similarity applies to propagation speed as well. The similarity between different cloud altitudes, as well as with high-altitude sprites, provides further support for the conclusion that the fast positive breakdown is streamer based (see Supplementary Note 1 for Additional Information).

What makes the fast positive breakdown unusual is not just its high speed, but also its strong VHF radiation. The latter is unexpected, since slowly propagating (≃10^5^ m s^−1^) positive streamers and leaders are RF faint and usually not detectable. An important question is how the VHF radiation is produced. While smoothly propagating positive streamer tips will not produce much VHF radiation, the fast breakdown would likely have transiently conducting tails in its wake that would be subject to substantial electric forces, making them good radiators of any instabilities that might occur.

Another issue is how the electric field reaches the point at which the fast breakdown is initiated. For hydrometeor-initiated streamers, the required field strength is estimated by Petersen *et al.*^32^ to be at least a factor of two greater than the largest electric fields observed in storms, measured to be ≃1–4 × 10^5^ V m^−1^ (refs 45, 46). While reviewing several means by which runaway electron avalanches could intensify the fields, Petersen *et al.* were careful to note that compact regions of locally strong electric fields could exist in storms that have eluded detection by *in situ* measurements. In the absence of observational evidence for such regions, however, the issue was considered to be unresolved. This led them to propose a hybrid mechanism in which runaway breakdown provided the field intensification needed for hydrometeor-initiated positive streamer systems.

There is still little or no evidence that energetic electron avalanches are involved in the initiation process, either in intensifying the electric field before a NBE or as causing the NBEs themselves. The most promising mechanism has been that proposed by Dwyer^47^, in which upward avalanching of energetic cosmic-ray background electrons develops retrogressively downward in the storm, substantially enhancing the electric field along the descending front of the avalanching. While the Dwyer mechanism would be an attractive way of enhancing the electric field to the point of initiating positive streamer breakdown^48^, the prerequisite avalanching leading up to the intensification should be readily detectable, but remains to be seen. Instead, observational evidence for locally intense electric field regions is found in the occurrence of short-duration precursor discharges in storms. In essence, precursor events serve as sensitive detectors of locally strong electric field regions.

Finally, we note that laboratory studies by Petersen *et al.*^49^ showed that individual positive streamers can be initiated by ice crystals in sub-dielectric breakdown conditions and at the low temperatures of storm altitudes. Recent modelling studies by Liu *et al.*^50^ have shown how the positive streamers would be initiated, both at low pressures above storms where sprites form, and at higher pressures to simulate hydrometeor initiation. In both cases, and in other experimental studies (for example, ref. 51) positive streamers are found to be much more easily initiated than negative streamers. Liu *et al.*,'s study showed that hydrometeor-initiated discharges start with a positive streamer that develops away from one end of the simulated hydrometeor, with negative activity either not occurring or being restricted to a localized region at the opposite end. The present results are consistent with this basic asymmetry for a larger-scale system of breakdown events. Liu *et al.*, specifically recognized the potential application of their results to the lightning initiation question (Supplementary Note 2).

## Methods

### Sferic simulations

The analytic expression for the double-exponential current waveform is given by:


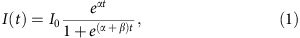


where *α*=1/*τ*_1_ and *β*=1/*τ*_2_ correspond to the rise and fall time constants of the current, assumed to be similar to those of the VHF radiation (for example, Supplementary Fig. 1). The propagation speed *v* was initially estimated from the change in elevation angle versus time, assuming vertical propagation. The overall amplitude was assumed to decrease exponentially with distance *z* along its propagation path, with an independently estimated 1/*e* distance constant *λ*. As seen in Supplementary Fig. 3, the current is assumed to turn on at the starting point of the NBE, to be slightly attenuated as it propagates, and to go to zero upon reaching the assumed end of the breakdown. The peak current *I*_pk_ is some fraction of *I*_0_, depending on the time constants. The induction and electrostatic components of the simulated electric field changes were determined using Equation A.38 of Uman^52^. The radiation component was determined using equation (11) of Shao *et al.*^53^ Two independent computational programs (at LANL and NM Tech) were used to check the validity of the simulations.

A key feature in interpreting the sferic waveforms was the positive-going partial field recovery seen between 21 and 24 μs in the NBE3 simulation of Fig. 2d. Due to the NBE being at close distance (3.3 km), its electric field change leading up to the recovery is dominated by the induction component, indicative of the breakdown current and its spatial moment^52^. Reproducing the field recovery indicates (indeed, requires) that the induction component rapidly decreased beyond the start of the recovery. By implication the current rapidly decreased as well. The partial field recovery provided an independent measure of the breakdown's extent. Assuming constant velocity, the extent is given by Δ*z*=*v*Δ*t*, where Δ*t* is the difference between the start times of the NBE and the field recovery (≃16 μs for NBE3). Using this, the simulation estimate of the NBE3's length is *z*_0_≃560 m, compared with the estimated vertical extent of ≃500 m from the INTF elevation observations. Beyond *z*_0_, the breakdown is indicated as having died out.

A similar field recovery began at 19 μs in the sferic for NBE1 (Fig. 2c, 13 μs into the NBE itself). The sferic waveform at this time showed a subtle but clear slope change that was again associated with current cessation. The recovery was slower than for NBE3, due to the current pulse being more lengthy, both temporally and spatially (Supplementary Fig. 3). In addition, the induction component was weaker due to NBE1's slightly greater plan distance (5.5 km versus 3.3 km), which in turn weakened the undershoot. The sensitivity of the induction component to plan distance is due to electrostatic and inductive field changes of dipolar discharges reversing polarity with increasing distance^12^. For short vertical discharges, the reversal distance 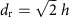
≃8.5 km for the NBEs of this study (*h*≃6 km being the height above approximate ground level in the mountainous terrain). This further emphasizes the importance of close measurements, as the induction and electrostatic components vanish for events near the reversal distance and decrease rapidly with range before and after that. (Because of NBE2's more complex activity, it is not clear when its current ceased, as the sferic did not exhibit a clear partial field recovery.)

The simulations were not overly sensitive to the attenuation rate *λ*, which emulated charge deposition along the breakdown path and only somewhat affected the shape of the simulated sferic. The attenuation was relatively weak, reducing the current amplitude only by a factor of two or so. This contrasts with previous NBE simulations for runaway electron avalanches^6^, for which the current increased exponentially with propagation distance, by a factor of 300–1,000 over similar path lengths (600–800 m).

In addition to the close sferics being well fitted by the simulations, measurements of the distant sferics were equally well fitted, in this case by the radiation component of the simulations (Supplementary Fig. 14). The comparisons were made utilizing the propagation code developed by Shao and Jacobson^54^ that accounts for the finite conductivity of the earth. In addition to being well simulated, the distant sferics confirm that the NBE breakdown was vertical rather than horizontal, as the sferics of horizontal breakdown do not survive propagating long distances.

### INTF and LMA measurements

The Langmuir broadband INTF was operated at 3,163-m altitude in the Magdalena mountains of central New Mexico. It measured the time derivative d*E*/d*t* of the VHF radiation at three flat-plate sensing antennas separated by 16 m in orthogonal horizontal directions. Time series data from each antenna were received over a 60-MHz bandwidth between 20 and 80 MHz, digitized at 180 MHz rate with 16-bit resolution and continuously streamed into computer memory, with a sufficient pretrigger record length (5 s) to capture entire flashes from post-flash triggering. The records typically began 2–3 s before flash onset, purposely allowing any precursor activity also to be captured. The effective dynamic range of the digitization was ≃75 dB. The resulting time series data were post-processed using generalized cross-correlation to determine the two-dimensional azimuth and elevation of the VHF radiation sources versus time, with 0.7–1.4-μs time resolution^9^. All three baselines were used for the processing, including the 22.6-m diagonal baseline of the right-triangle antenna array. The angular uncertainty of the observations depends on the signal-to-noise ratio and approached 0.1° r.m.s. for strong NBE sources between 30° and 60° elevation^9^. This corresponds to ≃12 m r.m.s. location uncertainty for sources at 7-km slant range, typical of the three high-power NBEs of this study. Empirically determined uncertainties of the elevation and azimuth source locations are presented in Supplementary Figs 15–20 for each of the events of the study, based on the redundant information obtained from using all three baselines (Supplementary Fig. 15 and Fig. 3.11 of Stock.^37^).

The LMA determined the source locations of impulsive radiation events in three spatial dimensions and time from 18 time-of-arrival measurement stations over a 50–65-km diameter area around and atop Langmuir Laboratory. The arrival times were measured with 35 ns r.m.s. uncertainty, corresponding to location uncertainties as small as 6–12 m r.m.s. in plan location and 10–20 m r.m.s. in altitude for non-noise contaminated sources^8^. The indicated VHF source powers correspond to peak values in the 60–66 MHz passband of the LMA^55^ and range from −25 dBW (3 mW) to greater than +60 dBW (1 MW). The data were recorded in successive 10-μs time intervals (windows) and decimated to 80-μs windows for the present analyses. The data were post-processed using a two-pass procedure, initially for events with (uncorrected) *χ*_ν_^2^ goodness of fit values less than unity, then up to 500 to ensure capturing the NBEs. This procedure produced a step decrease in the number of detected sources above unity *χ*_ν_^2^. The processing and resulting *χ*_ν_^2^ values assumed a nominal 70 ns r.m.s. timing uncertainty, but the empirically determined uncertainty of the network was 35 ns r.m.s. Thus, an indicated *χ*_ν_^2^=1 value corresponds to an actual value of (70/35)^2^ or 4.0. The *χ*_ν_^2^ values in this study are the indicated rather than corrected values, for uniformity of comparison with other networks and network configurations.

### Fast electric field measurements

The fast electric field change sensor utilized a flat-plate antenna and an integrating charge amplifier to measure *E*(*t*) directly. A fourth channel of the streaming digitiser recorded its measurements with the same wide dynamic range and bandwidth. The charge amplifier had a 100 μs decay time constant, which was accurately compensated for over short (≃100 μs) time intervals using standard deconvolution^56^ to ‘de-droop' the digitized data^57^. Matlab code that implements the algorithm for the simple RC exponential decay of the electric field change sensor is given by (R. G. Sonnenfeld, personal communication)


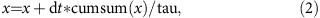


where *x* is the data array to be deconvolved, d*t* is the data sampling interval and tau is the decay time constant, corresponding to 1/180 MHz and 100 μs, respectively. Dedrooping was done only for the FA data used in the quantitative NBE simulations (Fig. 2; Supplementary Fig. 5). It had little effect on the recorded sferic for NBEs 1 and 2, but increased the electrostatic offset of NBE3 from −6 to −9 V m^−1^. The *E*(*t*) values were calibrated amplitude wise by comparing measured sferics for a number of intermediate-distance negative CG return strokes with peak current values and locations reported by the National Lightning Detection Network. The resulting conversion factor was ±32 K digital bits corresponded to ±30 V m^−1^ electric field change.

### Metric analyses

The basic idea behind the LMA analyses was the empirical finding from the 5 August observations that the very initial source of NBE-initiated discharges had a VHF power that was stronger than sources within 100 μs or so afterward—often noticeably stronger. Also, the time-of-arrival measurements for the initial source were often (but not necessarily) poorly fitted as well, as indicated by large least-square goodness of fit (*χ*_ν_^2^) values and incorrect source locations. The poor fits are due to the radiation being continuous and highly noisy for a microsecond or so around the time of its peak amplitude, often causing different LMA stations to detect slightly different peaks, which need to be correlated within ≃40 ns for a good least-square fit. That the LMA consistently detected the very initial VHF radiation source is supported by the overall observational data and by the sensitivity and quality of the network, routinely detecting events down to −20 or −25 dBW (3–10 mW) VHF power in the storms. Indeed, the LMA usually detected the first activity before the INTF did, particularly for more distant or weakly initiated flashes.

## Additional information

**How to cite this article:** Rison, W. *et al.* Observations of narrow bipolar events reveal how lightning is initiated in thunderstorms. 7:10721 doi: 10.1038/ncomms10721 (2016).

## Supplementary Material

Supplementary Information Supplementary Figures 1-20, Supplementary Notes 1-2 and Supplementary References.

## References

[b1] Smith D. A. *et al.* A distinct class of isolated intracloud lightning discharges and their associated radio emissions. J. Geophys. Res. 104, 4189–4212 (1999).

[b2] Rison W., Thomas R. J., Krehbiel P. R., Hamlin T. & Harlin J. A. GPS-based three-dimensional lightning mapping system: initial observations in central New Mexico. Geophys. Res. Lett. 26, 3573–3576 (1999).

[b3] Le Vine D. M. Sources of the strongest RF radiation from lightning. J. Geophys. Res. 85, 4091–4095 (1980).

[b4] Willett J. C., Bailey J. C. & Krider E. P. A class of unusual lightning electric field waveforms with very strong high-frequency radiation. J. Geophys. Res. 94, 16255–16267 (1989).

[b5] Eack K. B. Electrical characteristics of narrow bipolar events. Geophys. Res. Lett. 31, L20102 (2004).

[b6] Watson S. S. & Marshall T. C. Current propagation model for a narrow bipolar pulse. Geophys. Res. Lett. 34, L04816 (2007).

[b7] Liu H., Dong W., Wu T., Zheng D. & Zhang Y. Observation of compact intracloud discharges using VHF broadband interferometers. J. Geophys. Res. 117, D01203 (2012).

[b8] Thomas R. J. *et al.* Accuracy of the Lightning Mapping Array. J. Geophys. Res. 109, D14207 (2004).

[b9] Stock M. G. *et al.* Continuous broadband digital interferometry of lightning using a generalized cross-correlation algorithm. J. Geophys. Res. 119, 3134–3165 (2014).

[b10] Weiss S. A., Rust W. D., MacGorman D. R., Bruning E. C. & Krehbiel P. R. Evolving complex electrical structure of the STEPS 25 June 2000 multicell storm. Mon. Weather Rev. 136, 741–756 (2008).

[b11] Krehbiel P. R. The Electrical Structure Of Thunderstorms in The Earth's Electrical Environment National Academy Press (1986).

[b12] MacGorman D. R. & Rust W. D. The Electrical Nature of Storms Oxford Univ. Press (1998).

[b13] Nag A. & Rakov V. A. Compact intracloud lightning discharges: 1. Mechanism of electromagnetic radiation and modeling. J. Geophys. Res. 115, D20102 (2010).

[b14] Phelps C. T. Positive streamer system intensification and its possible role in lightning initiation. J. Atmos. Terr. Phys. 36, 103–111 (1974).

[b15] Griffiths R. F. & Phelps C. T. A model of lightning initiation arising from positive corona streamer development. J. Geophys. Res. 31, 3671–3676 (1976).

[b16] Shao X.-M. & Krehbiel P. R. The spatial and temporal development of intracloud lightning. J. Geophys. Res. 101, 26,641–26,668 (1996).

[b17] Winn W. P. *et al.* Lightning leader stepping, K changes, and other observations near an intracloud flash. J. Geophys. Res. 116, D23115 (2011).

[b18] Marshall T. *et al.* Initial breakdown pulses in intracloud lightning flashes and their relation to terrestrial gamma ray flashes. J. Geophys. Res. 118, 10,907–10,925 (2013).

[b19] Marshall T., Stolzenburg M., Karunarathna N. & Karunarathne S. Electromagnetic activity before initial breakdown pulses of lightning. J. Geophys. Res. 119, 12,558–12,574 (2014).

[b20] da Silva C. L. & Pasko V. P. Physical mechanism of initial breakdown pulses and narrow bipolar events in lightning discharges. J. Geophys. Res. 120, 4989–5009 (2015).

[b21] Marshall T. C. *et al.* Observed electric fields associated with lightning initiation. Geophys. Res. Lett. 32, L03813 (2005).

[b22] Shao X.-M., Krehbiel P. R., Thomas R. J. & Rison W. Radio interferometric observations of cloud-to-ground lightning phenomena in Florida. J. Geophys. Res. 100, 2749–2783 (1995).

[b23] Krehbiel P. R. *et al.* Upward electrical discharges from thunderstorms. Nat. Geosci. 1, 233–237 (2008).

[b24] Defer E. *et al.* Lightning activity for the July 10, 1996, storm during the Stratosphere-Troposphere Experiment: Radiation, Aerosol, and Ozone-A (STERAO-A) experiment. J. Geophys. Res. 106, 10,151–10,172 (2001).

[b25] Rison W., Krehbiel P. R., Thomas R. J. & Rodeheffer D. Lightning mapping observations of volume-filling small discharges in thunderstorms. AGU Fall Annual Meeting, Abstract AE13A-0339 (2013).

[b26] Bruning E. C., Rust W. D., MacGorman D. R., Biggerstaff M. I. & Schurr T. J. Formation of charge structures in a supercell. Mon. Weather Rev. 138, 3740–3761 (2010).

[b27] Mazur V. Rapidly occurring short duration discharges in thunderstorms, as indicators of a lightning-triggering mechanism. Geophys. Res. Lett. 13, 355–358 (1986).

[b28] Rison W., Krehbiel P. R., Thomas R. J. & Brown M. F. Observations of precursor breakdown prior to intracloud lightning discharges. Eos Trans. AGU 90, Fall Meeting Suppl., Abstract AE32A-02 (2009).

[b29] Edens H. E., Krehbiel P. R. & Rison W. VHF lightning mapping observations of screening charge flashes at thunderstorm tops. AGU Fall Annual Meeting, Abstract AE22A-01 (2014).

[b30] Edens H. E. Photographic and lightning mapping observations of a blue starter over a New Mexico thunderstorm. Geophys. Res. Lett. 38, L17804 (2011).

[b31] Wu T., Yoshida S., Ushio T., Kawasaki Z. & Wang D. Lightning-initiator type of narrow bipolar events and their subsequent pulse trains. J. Geophys. Res. 119, 7425–7438 (2014).

[b32] Petersen D., Bailey M., Beasley W. & Hallett J. A brief review of the problem of lightning initiation and a hypothesis of initial lightning leader formation. J. Geophys. Res. 113, D17205 (2008).

[b33] Kitagawa N. & Brook M. A comparison of intracloud and cloud-to-ground lightning discharges. J. Geophys. Res. 65, 1189–1201 (1960).

[b34] Loeb L. B. The mechanisms of stepped and dart leaders in cloud-to-ground lightning strokes. J. Geophys. Res. 71, 4711–4721 (1966).

[b35] Dawson G. A. & Winn W. P. A model for streamer propagation. Zeit. Phys. 183, 159–171 (1965).

[b36] Jacobson A. R., Light T. E. L., Hamlin T. & Nemzek R. Joint radio and optical observations of the most radio-powerful intracloud lightning discharges. Ann. Geophys. 31, 563–580 (2013).

[b37] Stock M. A. Broadband interferometry of lightning Ph.D. Thesis, New Mexico Institute Mining and Technology (2014).

[b38] Stock M. A., Krehbiel P. R., Rison W., LaPierre J. & Edens H. E. Observations of fast VHF-bright positive breakdown. AGU Fall Annual Meeting, Abstract AE22A-04 (2014).

[b39] Stanley M. *et al.* High speed video of initial sprite development. Geophys. Res. Lett. 26, 3201–3204 (1999).

[b40] Cummer S. A. *et al.* Submillisecond imaging of sprite development and structure. Geophys. Res. Lett. 33, L04104 (2006).

[b41] Luque A. & Ebert U. Emergence of sprite streamers from screening-ionization waves in the lower ionosphere. Nat. Geosci. 2, 757–760 (2009).

[b42] Briels T. M. P., van Veldhuizen E. M. & Ebert U. Positive streamers in air and nitrogen of varying density: experiments on similarity laws. J. Phys. D Appl. Phys. 41, 234008 (2008).

[b43] Pasko V. P. Red sprite discharges in the atmosphere at high altitude: the molecular physics and the similarity with laboratory discharges. Plasma Sources Sci. Technol. 16, S13–S1329 (2007).

[b44] Ebert U. & Sentman D. D. Streamers, sprites, leaders, lightning: from micro- to macroscales. J. Phys. D Appl. Phys. 41, 230301 (2008).

[b45] Winn W. P., Schwede G. W. & Moore C. B. Measurements of electric fields in thunderclouds. J. Geophys. Res. 79, 1761–1767 (1974).

[b46] Stolzenburg M. *et al.* Electric field values observed near lightning flash initiations. Geophys. Res. Lett. 34, L04804 (2007).

[b47] Dwyer J. R. The initiation of lightning by runaway air breakdown. Geophys. Res. Lett. 32, L20808 (2005).

[b48] Dwyer J. R. & Uman M. A. The physics of lightning. Phys. Rep. 534, 147–241 (2014).

[b49] Petersen D., Bailey M., Hallett J. & Beasley W. H. Laboratory investigation of positive streamer discharges from simulated ice hydrometeors. Q. J. R. Meteorolog. Soc. 132, 263–273 (2006).

[b50] Liu N., Kosar B., Sadighi S., Dwyer J. R. & Rassoul H. K. Formation of streamer discharges from an isolated ionization column at subbreakdown conditions. Phys. Rev. Lett. 109, 025002 (2012). 23030169 10.1103/PhysRevLett.109.025002

[b51] Briels T. M. P., Kos J., Winands G. J. J., van Veldhuizen E. M. & Ebert U. Positive and negative streamers in ambient air: measuring diameter, velocity and dissipated energy. J. Phys. D Appl. Phys. 41, 234004 (2008).

[b52] Uman M. A. The Lightning Discharge Academic Press (1987).

[b53] Shao X.-M., Fitzgerald T. J. & Jacobson A. R. Reply to comment by Rajeev Thottappillil and Vladimir A. Rakov on ‘Radio frequency radiation beam pattern of return strokes: a revisit to theoretical analysis.'. J. Geophys. Res. 110, D24106 (2005).

[b54] Shao X.-M. & Jacobson A. R. Model simulation of very-low-frequency and low-frequency lightning signal propagation over intermediate ranges. IEEE Trans. Electromag. Compat. 51, 519–525 (2009).

[b55] Thomas R. J. *et al.* Observations of VHF source powers radiated by lightning. Geophys. Res. Lett. 28, 143–146 (2001).

[b56] Mitra S. K. Digital Signal Processing: a Computer-Based Approach McGraw Hill (2011).

[b57] Holmes C. R., Szymanski E. W., Szymanski S. J. & Moore C. B. Radar and acoustic study of lightning. J. Geophys. Res. 85, 7517–7532 (1980).

