# Supplementary material for: Observations of narrow bipolar events reveal how lightning is initiated in thunderstorms
Source: Nat Commun. 2016 Feb 15;7:10721. doi: 10.1038/ncomms10721 (PMC4756383; doi:10.1038/ncomms10721)
Supplement: Supplementary Information — Supplementary Figures 1-20, Supplementary Notes 1-2 and Supplementary References. [file ncomms10721-s1.pdf]

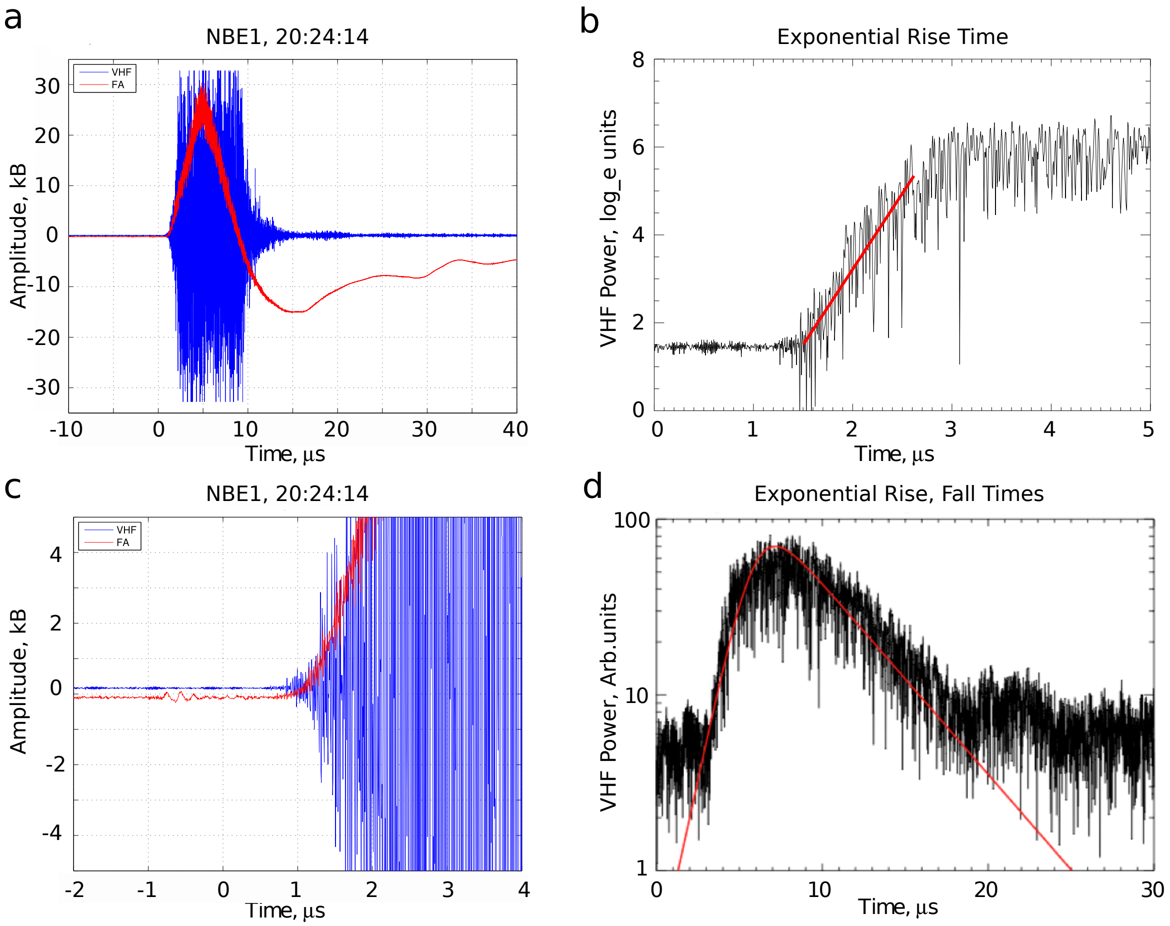

Supplementary Figure 1: Detailed time series data for NBE1. a, INTF (blue) and FA (red) waveforms for NBE1, showing clipping of the VHF signal by the digitiser. This did not affect the ability of the INTF to determine the source directions, as the phase of the signals was unchanged by the clipping. b, Exponential increase in the VHF power during the first 1.5 μs of the NBE, prior to being clipped. The red line corresponds to a 0.3 μs initial rise time constant. c, Expanded plot of the first few μs of the NBE, showing i) its rapid onset, ii) the simultaneous occurrence of the FA sferic and VHF radiation, indicative of the current being produced by the breakdown, and iii) the lack of detectable activity prior to the NBE onset. Both the VHF and fast electric field signals were quiet down to the ambient noise level of the INTF site, $\simeq$66 dB below their full scale amplitudes. The transient ripple in the FA waveform is due to periodic local interference source, seen in Fig. 4d and in Supplementary Fig. 2b. d, Exponential rise and fall of the VHF power obtained from the attenuated VHF waveform present on the FA signal, digitised at the same 180 MHz rate as the INTF signals. The overall rise and fall time constants were $\simeq$1.0 and 4.7 μs, respectively.

...

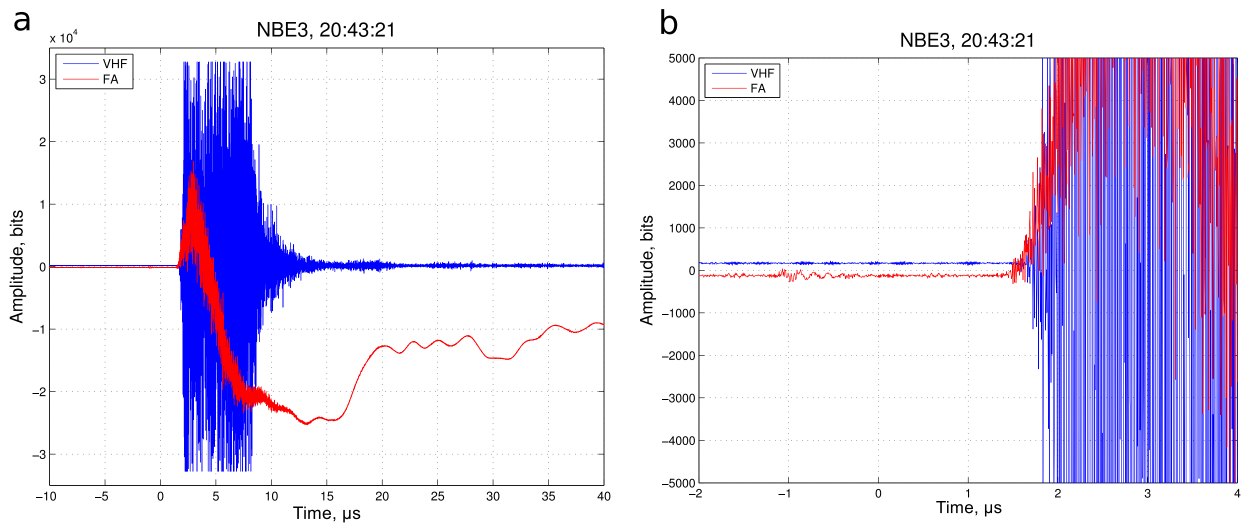

Supplementary Figure 2: Time series data for NBE3. a, Overview of INTF and FA waveforms. b, Expanded view of the first few microseconds of the NBE, showing its fast onset and lack of prior activity, similar to NBE1.

...

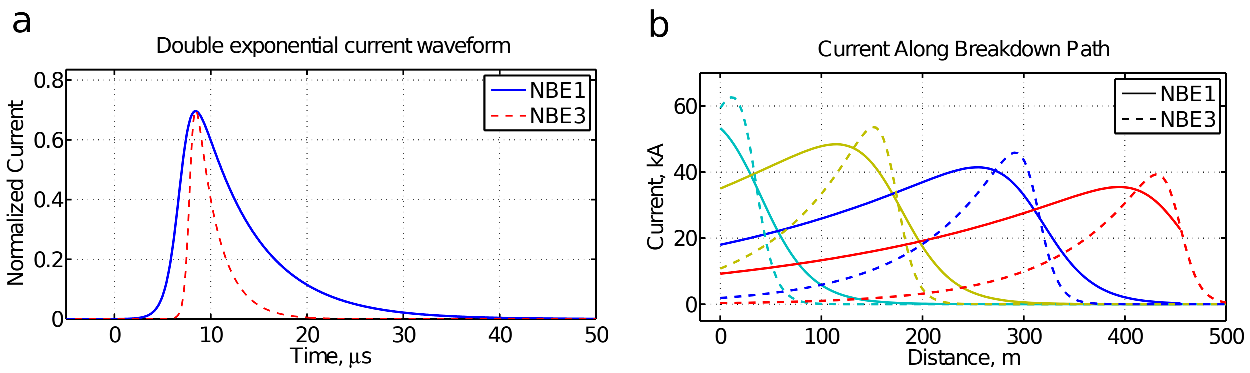

Supplementary Figure 3: Current waveforms. a, Double exponential current waveforms that simulated the sferics for NBE1 and NBE3, showing the difference in duration of the two currents. b, Current versus propagation distance at successive 4 μs time intervals, illustrating how the current for NBE3 was spatially more compact while the current for NBE1 appeared to be spatially spread out relative to the overall extent of the breakdown, consistent with the NBEs being produced by a succssion of breakdown events.

...

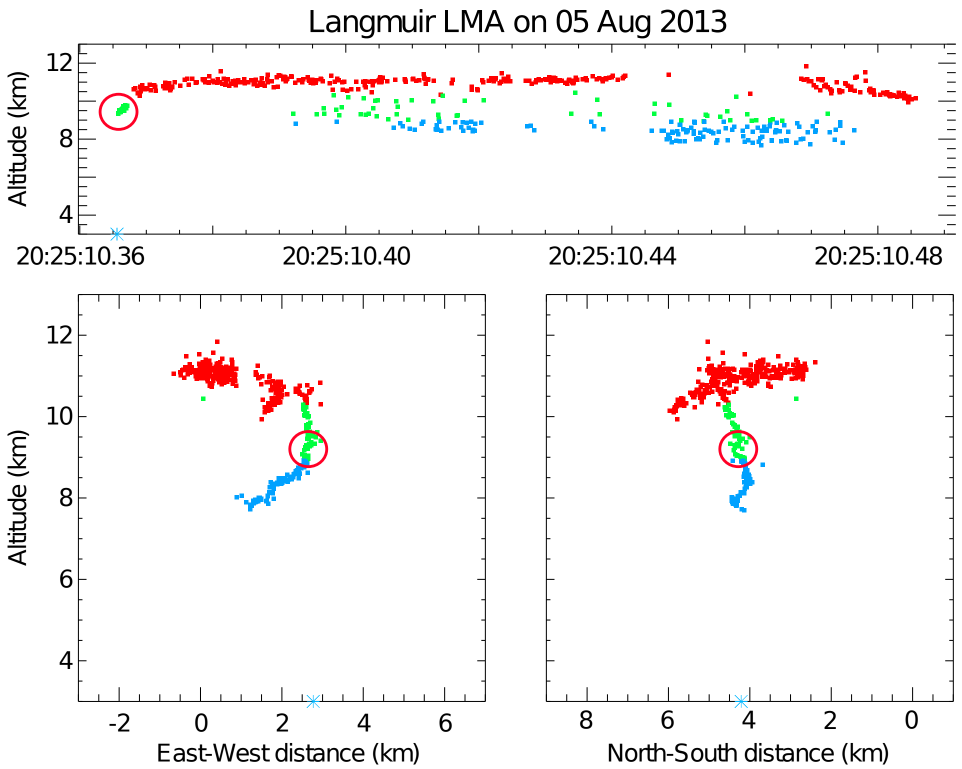

Supplementary Figure 4: LMA observations for the NBE2 flash. Same as Fig.1b, except showing the first 120 ms of the bilevel IC flash initiated by NBE2. Like NBE1, NBE2 (red circle) occurred at the base of the upward negative leader of the flash (green sources). The E-W and N-S vertical projections show that the negative leader was primarily vertical, consistent with the NBE also being vertical.

...

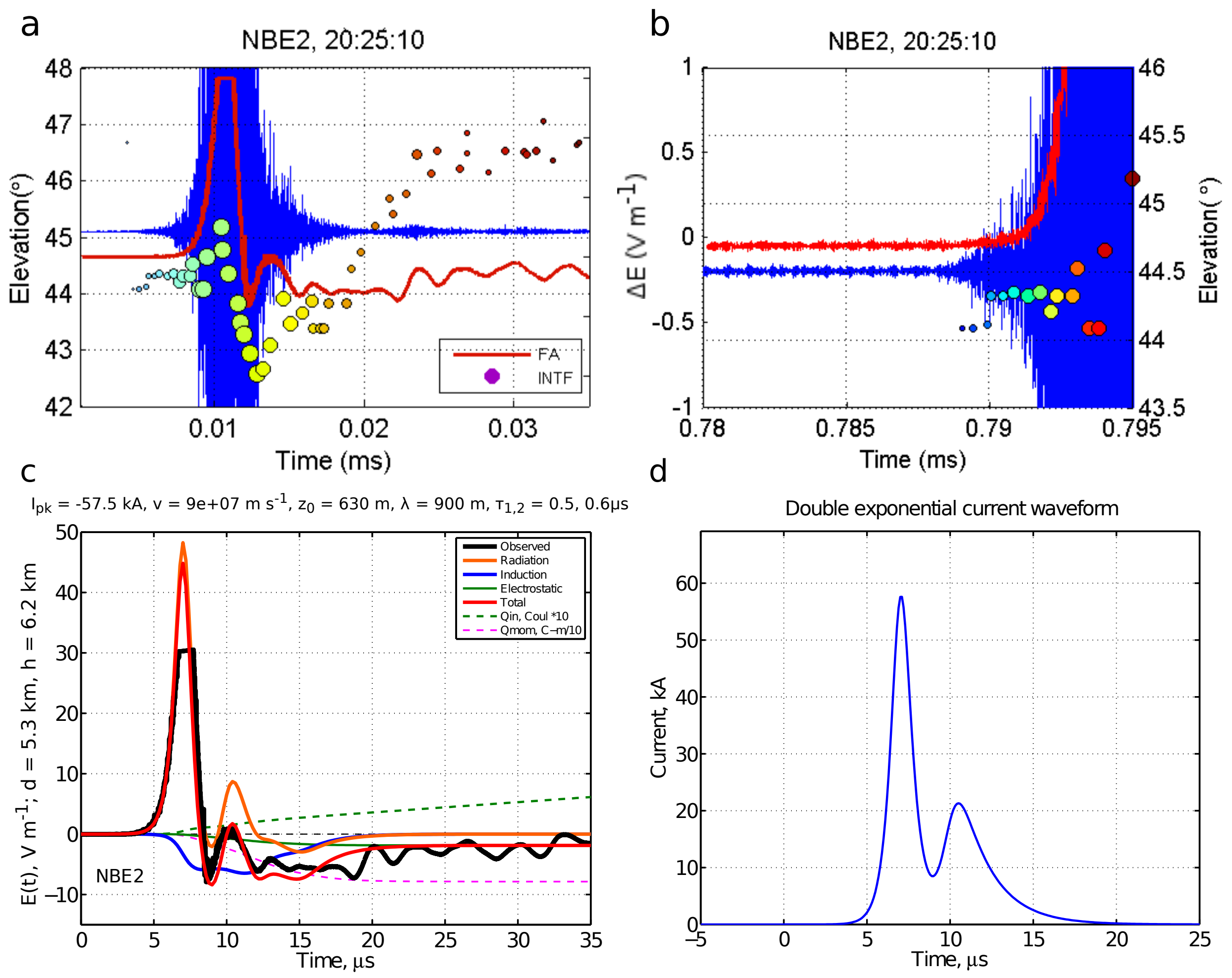

Supplementary Figure 5: Detailed observations for NBE2. a, Overview of the INTF and FA observations. b, Expanded view of the initial 7 μs of the NBE, showing i) the relatively gradual onset of the VHF activity, ii) the retrogressive upward development of the initial breakdown, iii) several attempted downward events as the discharge intensified, and iv) the lack of activity prior to the NBE's onset. c,d, simulation of NBE2's sferic and the two-pulse current waveform used in the simulation (see main text).

...

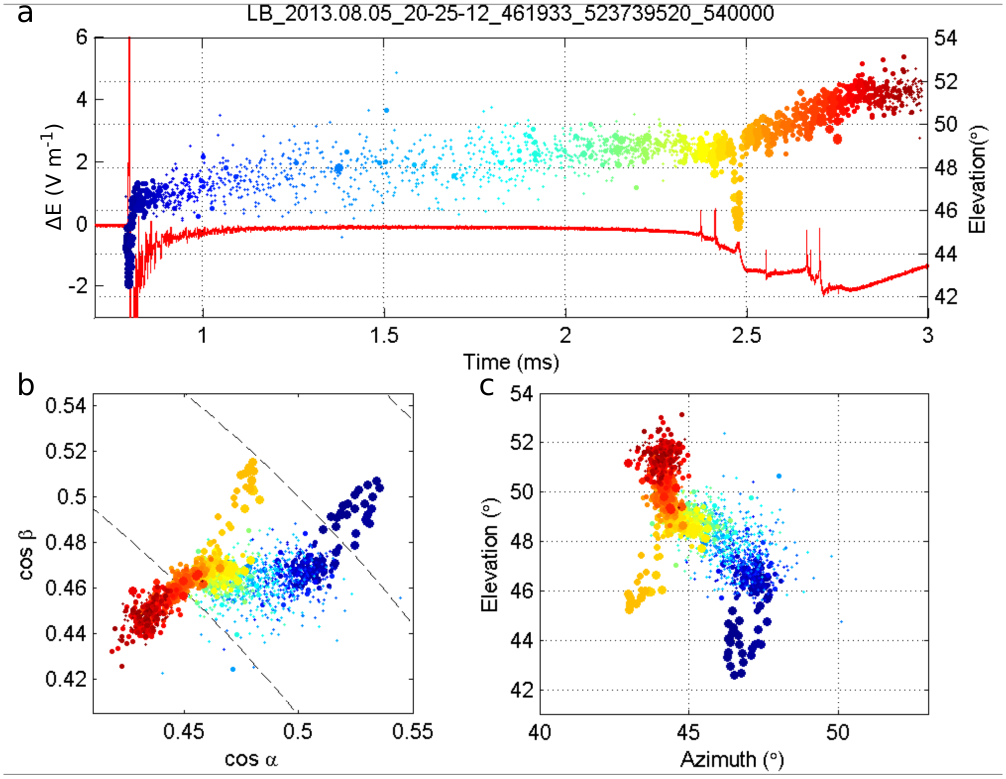

Supplementary Figure 6: Initial activity of the NBE2 flash. a, Detailed INTF and FA observations for the first 2 ms of the IC flash initiated by NBE2, illustrating the two-stage nature of the negative breakdown: i) gradual upward development during the 1.5 ms-duration initial electric field change (IEC), ii) the fine structure of the first initial breakdown pulse (IBP) following the IEC, iii) the occurrence of the second NBE (downward orange sources) at the beginning of the IBP, and iv) the accelerated upward development associated with the IBP. b,c, INTF source locations in the plan projection plane (Supplementary Fig. 15) and in azimuth-elevation format, showing the upward negative breakdown two-dimensionally. The IEC (blue and green sources) extended the discharge $\simeq$500 m at an estimated speed of $\simeq 3 \times 10^5$ m s^{-1}, and the IBP (yellow and red sources) extended the discharge a similar additional distance at $\simeq 1 \times 10^6$ m s^{-1}. In this and other figures, the INTF sources are coloured by time and and sized logarithmically by power.

...

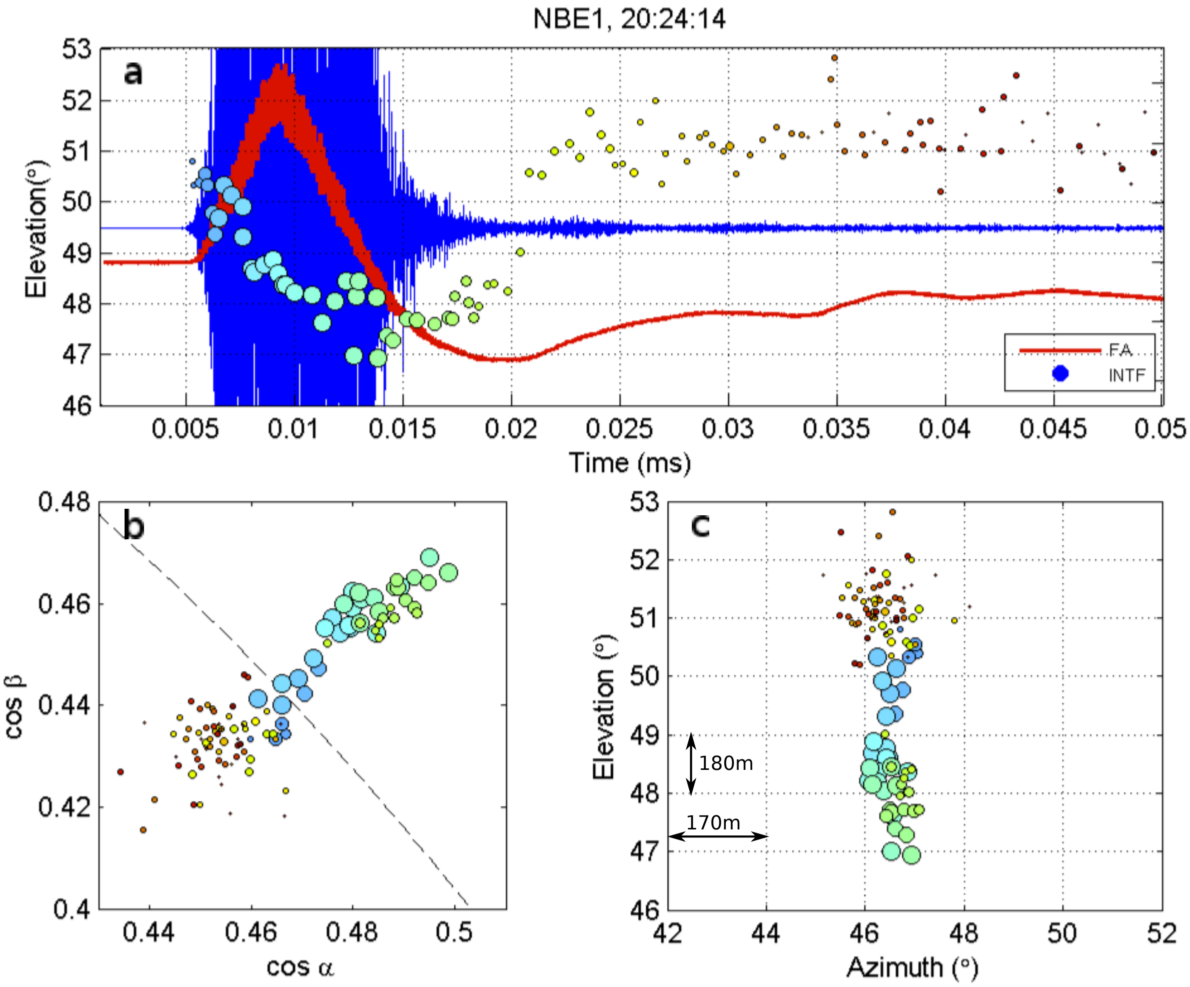

Supplementary Figure 7: Detailed INTF data for NBE1. a, First 50 μs of NBE1, as in Fig. 2a except expanded in time to better illustrate the altitude variability of the NBE VHF sources, indicative of a succession of breakdown events. b,c, Projection plane and azimuth-elevation plots of the VHF sources, showing i) that the NBE breakdown occurred at constant azimuth, consistent with the positive breakdown being vertically downward, and ii) that the radiation sources following the NBE were directly above the NBE start point, consistent with being produced by the onset of negative breakdown. The horizontal distance scale in panel c) indicates the azimuthal arc length at the plan distance of the discharge. The elevation scale corresponds to vertical distance rather than arc length. See Supplementary Fig. 16 for the uncertainty of the source locations.

...

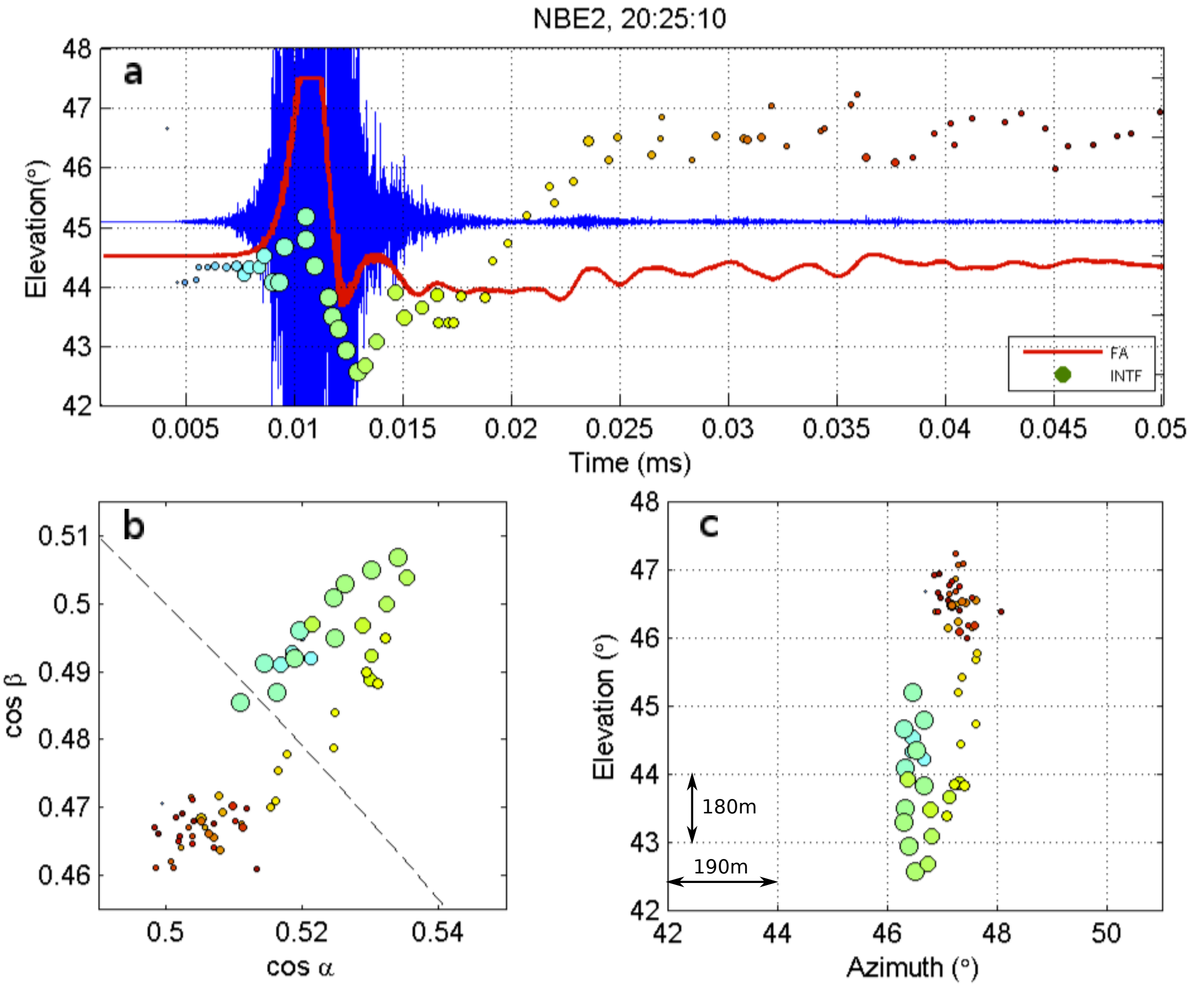

Supplementary Figure 8: Detailed INTF data for NBE2. a, Expanded view of first 50 μs of the flash (see also Fig. 3 and Supplementary Figs. 5 and 6). b,c, Projection plane and azimuth-elevation locations of the VHF sources. While the downward part of the NBE occurred at constant azimuth, the post-NBE sources associated with negative breakdown had already developed slightly away from the starting region of the NBE. See Supplementary Fig. 17 for the uncertainty in the INTF source locations.

...

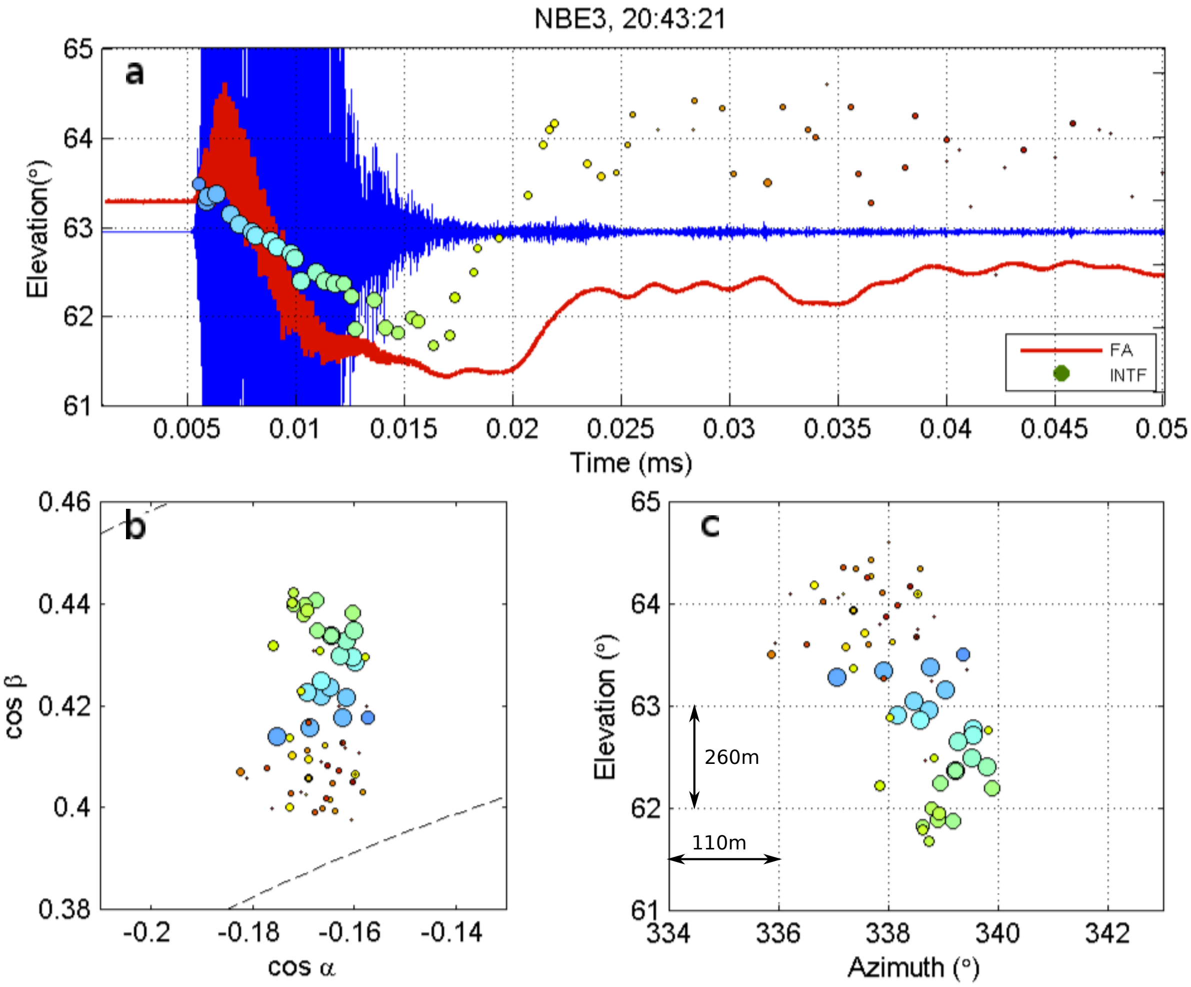

Supplementary Figure 9: Detailed INTF data for NBE3. a, Expanded time plot of the first 50 μs of the flash (Fig. 2b). b,c, Projection plane and azimuth-elevation locations of the VHF sources, showing substantial azimuthal spread in the VHF sources, both during and following the NBE, indicative of the positive breakdown being laterally distributed, and also tilted somewhat from vertical. See Supplementary Fig. 18 for the uncertainty in the INTF source locations.

...

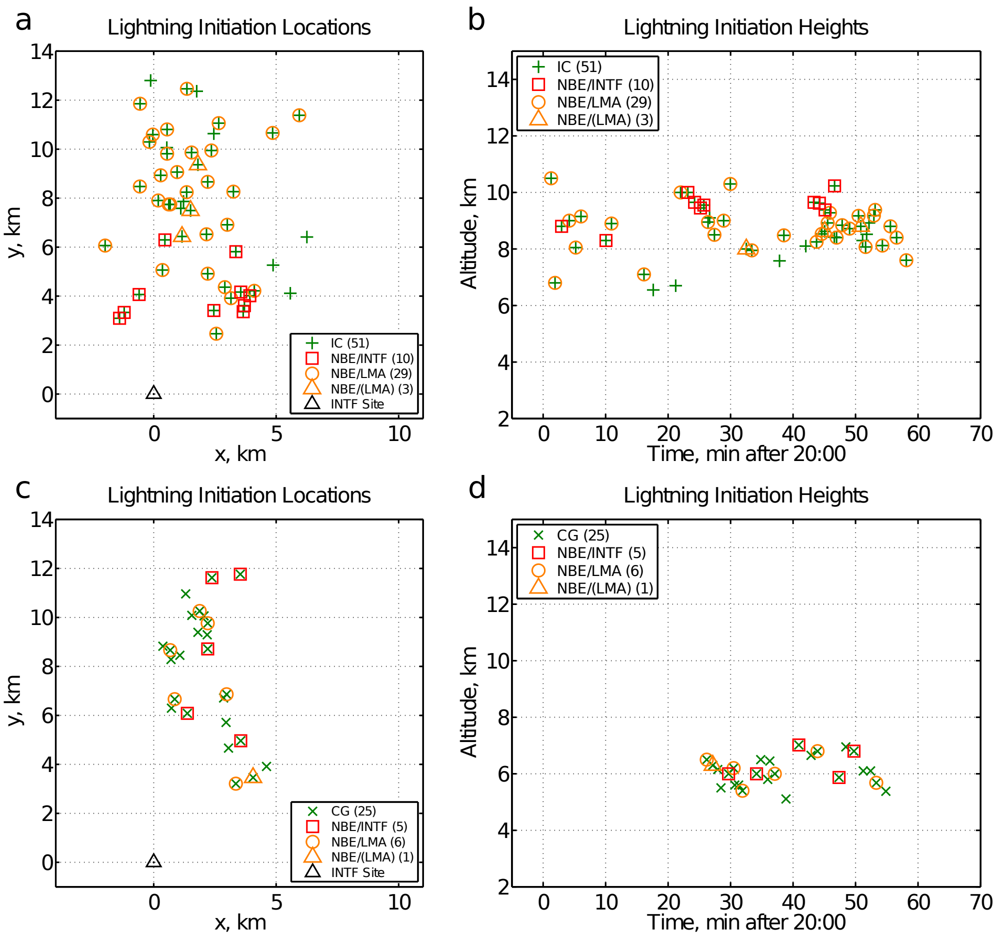

Supplementary Figure 10: Initial LMA sources for all lightning flashes in the 5 August NBE storm. (Supplement to Fig. 5) a,b, Plan locations and initiation altitudes vs. time for the IC flashes. c,d, Same, except for CG flashes. NBEs 1 and 2 occurred in the concentration of events 5–6 km immediately NE of the INTF (panel a). NBE3 occurred as the first and closest of three clustered IC flashes in a small subsequent cell that developed 3–4 km immediately NNW of the INTF. The plan location results show the tendency for IC flashes to have their initiation confirmed by INTF observations (red squares) when they were close to the INTF (black triangle), but this decreased with increasing distance, due largely to the INTF recording being triggered less often by ICs unless close. Due to the LMA's large deployment and coverage area, it detected IC and CG flashes equally well independent of location, allowing the initiation of many of the IC flashes to be identified from the LMA data alone (orange circles). By contrast, the monopolar electric field change of CG flashes enabled INTF recordings to be triggered for most of the CG flashes and to be used in their initiation determination. Of the 76 total flashes, 42 triggered the INTF recording, with 21 of 25 CG flashes being so recorded, compared to 21 of 51 IC flashes. Note the clear demarcation of the initiation altitudes of IC and CG flashes, with the ICs typically being initiated between 8–10 km MSL and negative CGs between 5.5–7 km. Several subsidence episodes occurred during the hour-long storm, during which time coupled altitude decreases were seen in the CG and IC initiation heights. The episode between 20 and 30 minutes was associated with intensfied lightning activity and the first two NBEs.

...

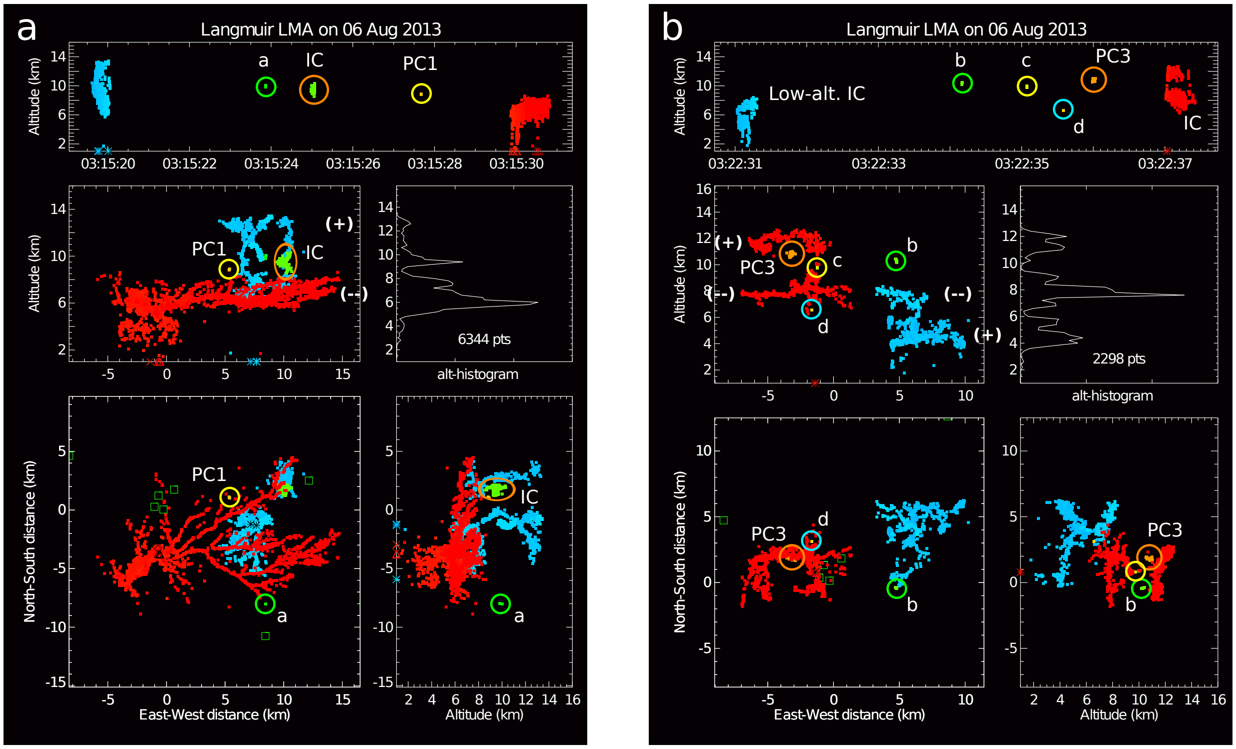

Supplementary Figure 11: Precursor events on 6 August. a, Lightning activity during a 10 s interval between 03:15:20 and 03:15:30, showing the occurrence of precursor PC1 of Fig. 6a (yellow circles) and two other short-duration discharges, in relation to the preceding and subsequent lightning flashes. b, Same, except between 03:22:31 and 03:22:37, showing the occurrence of PC3 of Fig. 6b (orange circles). These and the other circled PC events were captured in the preflash intervals of INTF recordings triggered by the subsequent flashes. PC1 occurred immediately above the periphery of a horizontally extensive CG flash that followed 2 s later (red sources). PC3 occurred 1 s before a bilevel IC flash (red sources), a short distance away from the IC's upward channel and immediately below upper positive charge discharged by the IC. Precursor 'd' was an uncommon low-altitude event that occurred 1.5 s before and immediately below mid-level negative charge discharged by the IC.

...

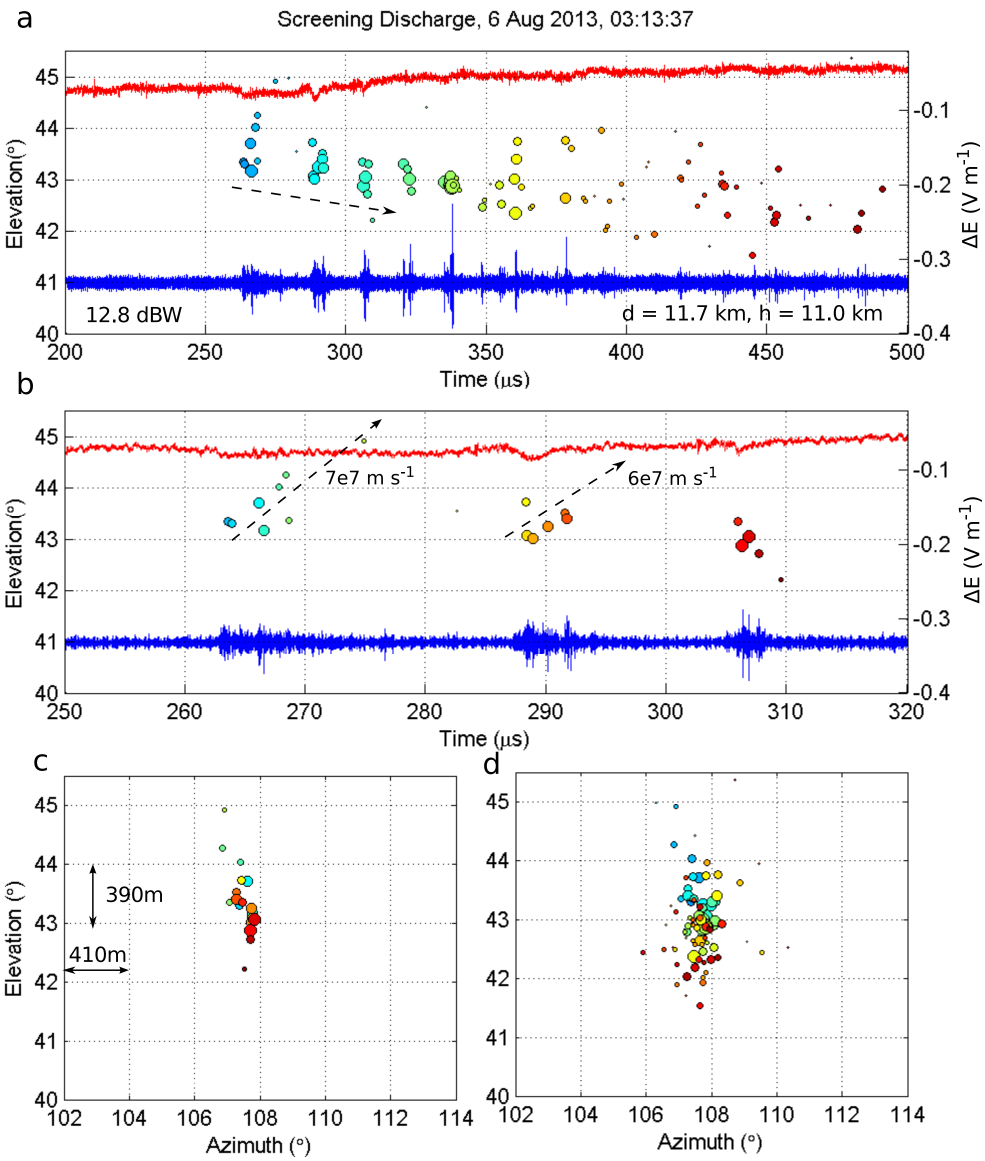

Supplementary Figure 12: Detailed observations of the screening discharge. a, Expanded time series data for the temporally-separated upward positive breakdown events at the beginning of the 6 ms-duration screening discharge of Fig. 6c, showing the retrogressive downward development of the successive events (dashed arrow). b, Additionally expanded data for the first three events, showing the apparent speeds of the first and second discharges. Each successive event had decreased vertical extent but slightly stronger peak VHF activity. c,d, Spatial development of the VHF radiation sources in azimuth-elevation format during the initial 50 and 250 μs of the discharge, respectively. Although the discharge was at 11.7 km plan distance, due to its great height (14.2 km), it was near 45° elevation angle relative to the INTF at 3.2 km MSL. Uncertainties in the INTF source locations are shown in Supplementary Fig. 20.

...

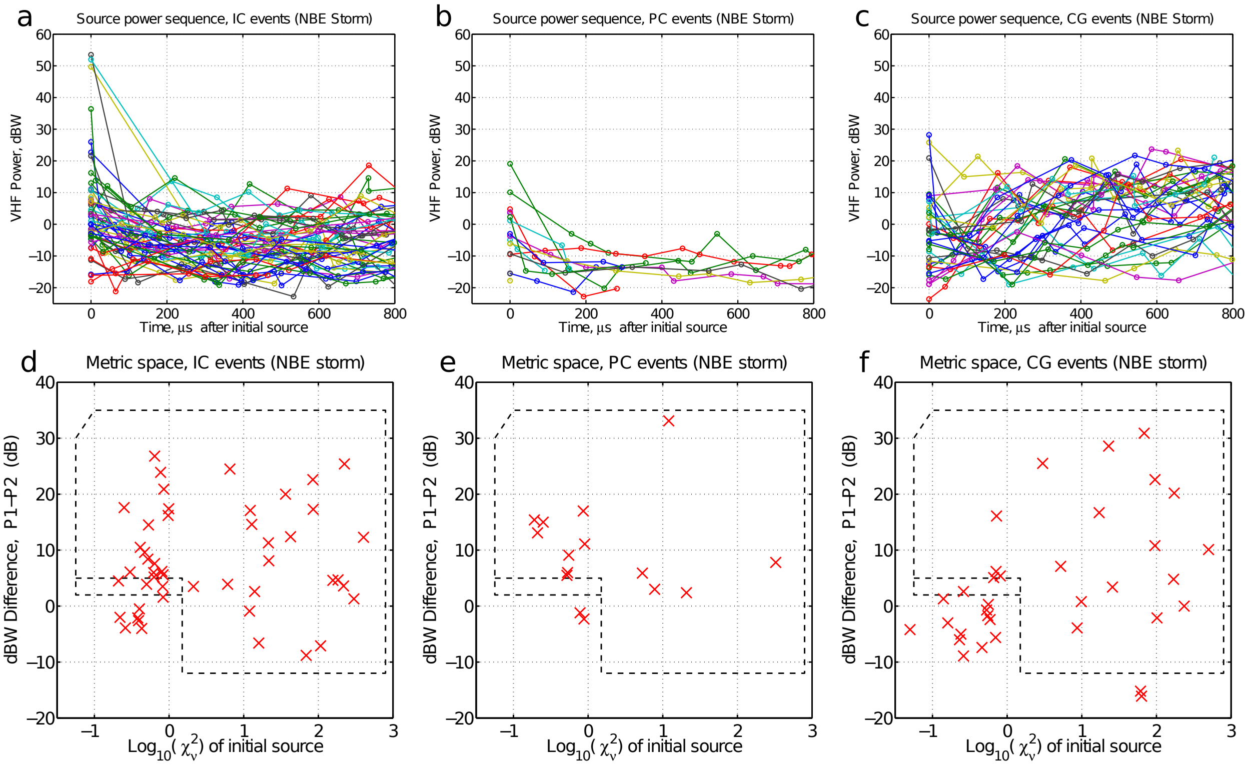

Supplementary Figure 13: Detailed analysis of LMA data for the 5 August 2013 flashes. a–c, VHF source power sequences for the first 800 μs of the IC, PC, and CG discharges (same as Fig. 7a–c for 6 August, except for the hour-long NBE storm). d–f, Same, except showing the observations in the metric space of Fig. 7d–f. Fewer PCs were produced by the NBE storm than during the 3.5 min interval of the 6 August storms, but the metrics indicated nearly all were NBE-initiated. Larger numbers of CG discharges had negative first and second source power differences ΔP, and thus were classified as being undetermined by the metric. As noted in connection with Fig. 7, this is likely an artifact of rapid intensification of the breakdown. A larger fraction of ICs were indicated as being NBE-initiated metric-wise.

...

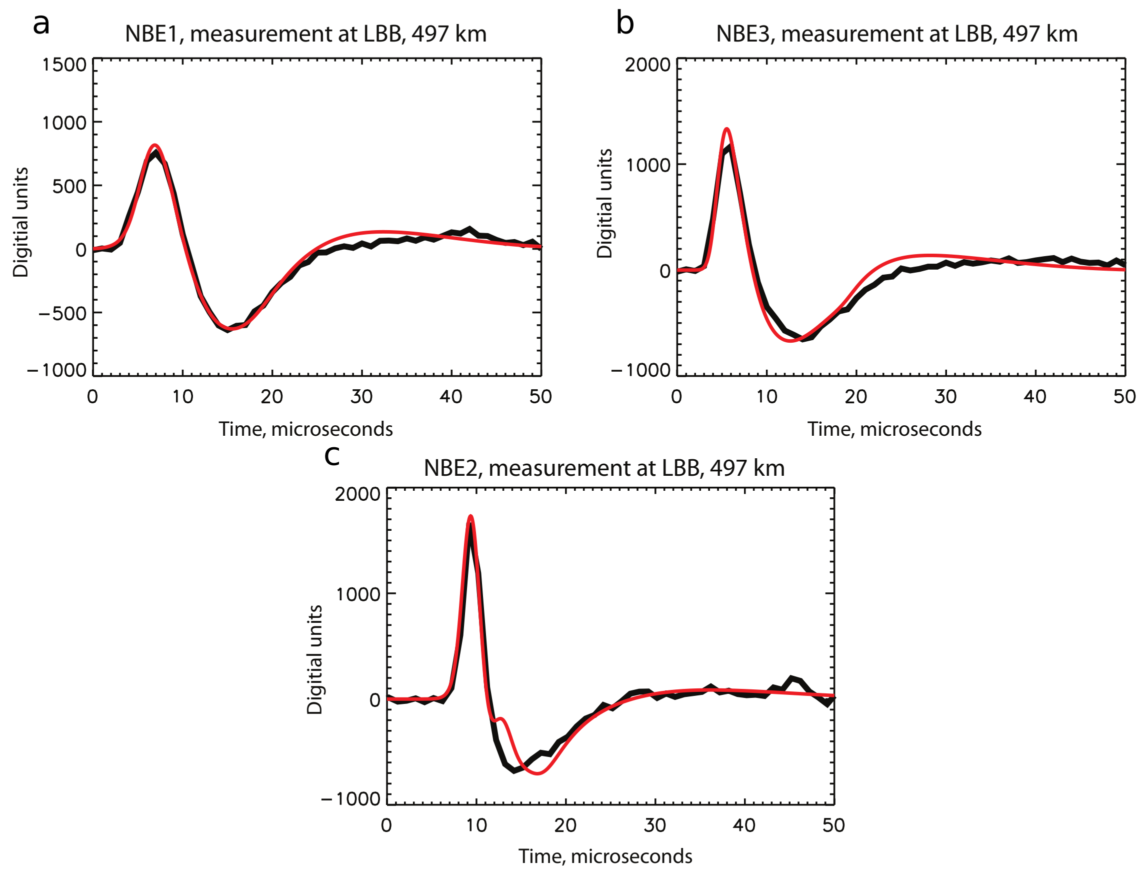

Supplementary Figure 14: Distant sferic comparisons. a–b, Comparison of the simulation-predicted (red) and observed (black) sferic for NBEs 1 and 3 (Fig. 2) at the 500-km distant Lubbock, Texas station of the Los Alamos Sferics Array (LASA),[1] documenting that the model-estimated radiation component for the NBEs is consistent with the distant sferic. The predicted sferic takes into account propagation effects over a finitely-conducting earth. The agreement indicates the NBEs were vertically oriented (horizontal breakdown would be strongly attenuated at the measurement distance). c, Same, except for NBE2, simulated in Supplementary Fig. 5. The secondary pulse of the NBE2 sferic is not seen at the Lubbock station. Its amplitude may be overestimated in the simulation or its cause not fully understood.

...

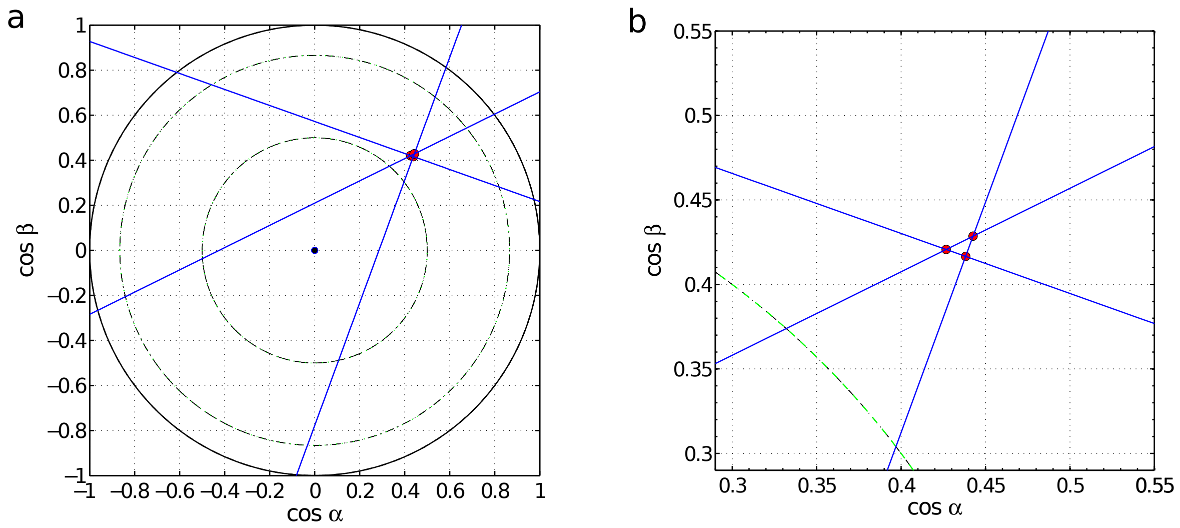

Supplementary Figure 15: INTF source location example. a, Direction cosine projection plane of the INTF observations, illustrating how the radiation sources are located. The arrival time differences between pairs of antennas constrains the source to lie along a straight lines in the direction cosine plane, perpendicular to the baselines.[2] The intersection of the lines gives the direction of the source. Geometrically, the direction cosine plane corresponds to the equatorial plane of a unit celestial hemisphere centered on the antenna array.[3,4] The inscribed circle corresponds to the horizon and its center to the zenith. The dashed circles correspond to 30° and 60° elevation angles and shows that the interferometer locates sources less well below 30° elevation. The azimuth and elevation angles are obtained by projecting the solutions in the direction cosine plane up onto the celestial hemisphere. b, Zoomed-in view of the intersection. Noise and/or multiple sources cause the lines not to have a common intersection, but to outline a triangular region. The solution is obtained from a least-square fit of the lines and lies within the triangle. The size of the triangle provides a measure of the uncertainty in the source direction. For the example shown, the time differences of arrival at the three baselines were $(\tau_{12}, \tau_{23}, \tau_{31}) = (+0.0294, -0.0152, -0.0149)$ μs. The maximum and minimum azimuth angles were 45.408° and 46.452° and the elevation angles were 51.968° and 53.187°, corresponding to 'peak-to-peak' uncertainties of 1.04° in azimuth and 1.22° in elevation around the least-square value. For point source and noise-free observations, the three time differences (i.e., the 'closure delay' τ_{123} going around the array) add to zero. For the example shown, the time differences add to $\tau_{123} = -0.0007$ μs, or –0.7 ns. The uncertainties provide a measure of the combined effects of noise and non-localized radiation sources. The observations indicate the uncertainty is primarily due to multiple sources rather than background noise (e.g, Supplementary Figs. 16 and 17).

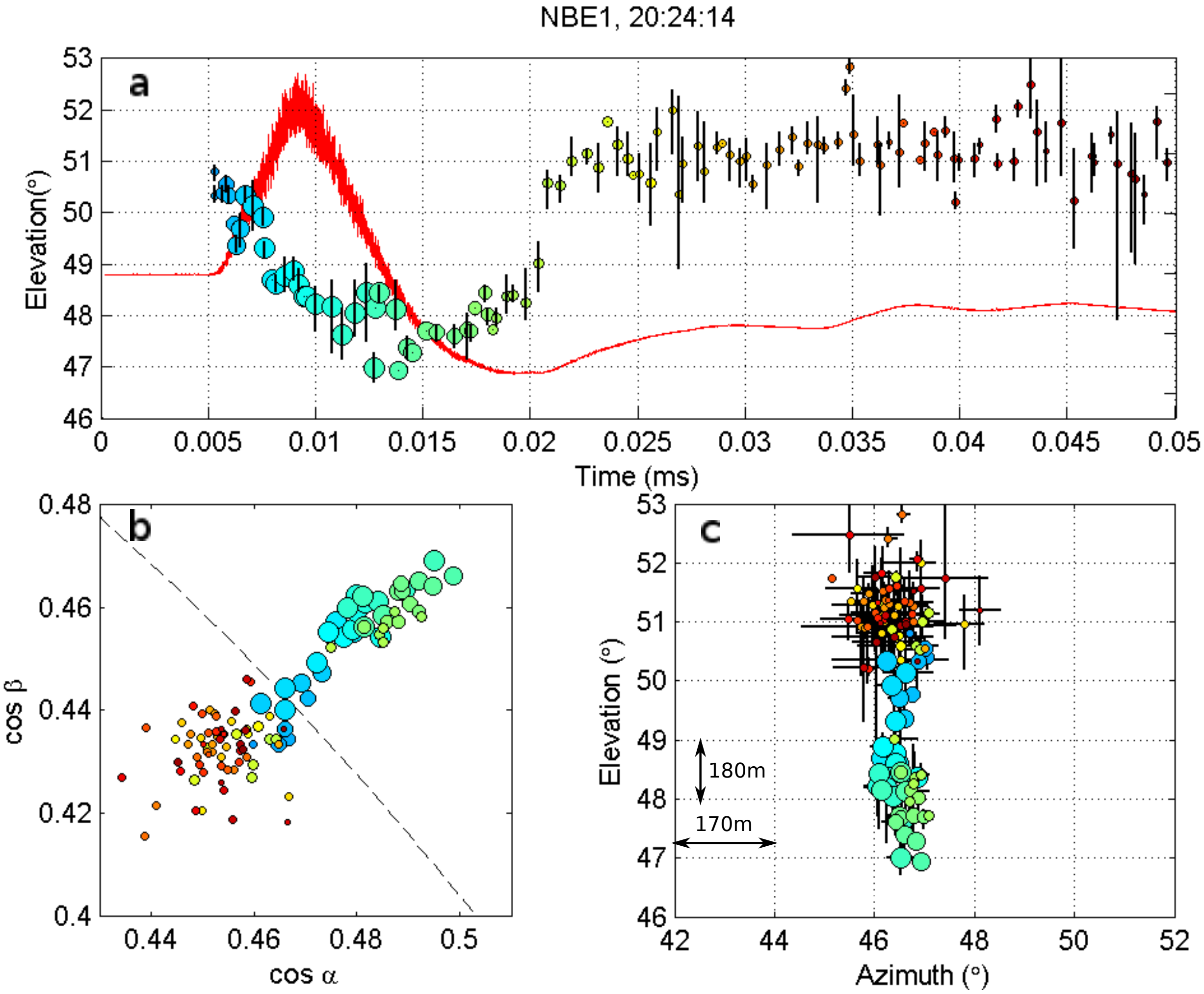

Supplementary Figure 16: Source location uncertainties for NBE1. Same as Supplementary Fig. 7, except showing the elevation and azimuth uncertainties of the VHF source locations (black vertical lines in panel a and crosses in panel c). For clarity, the uncertainties are overlaid on top of the VHF sources in panel a) and behind the sources in panel c). Sources with centered dots in panel a) have a small uncertainty. The noticeable increase in the elevation uncertainty following the NBE (panel a) and also in azimuth (panel c) is due primarily to the negative breakdown having multiple sources, rather than to their reduced power and signal to noise ratio. This is seen from the first two sources at the beginning of the NBE being as weak or weaker than the post-NBE activity, yet having very small elevation uncertainties ($\simeq$0.2–0.3°). It indicates the initial breakdown of the NBE was highly localized, and illustrates the limiting accuracy of the INTF measurements. The elevation uncertainties remain relatively small during the initial part of the downward NBE, but increase for some of the sources toward the end of the NBE, consistent with the activity occurring at multiple altitudes. The sudden elevation increase just after 0.02 ms (20 μs) is significant uncertainty-wise and coincides with the beginning of the partial field recovery, further supporting the result that the recovery signals the current having died out beyond this time.

...

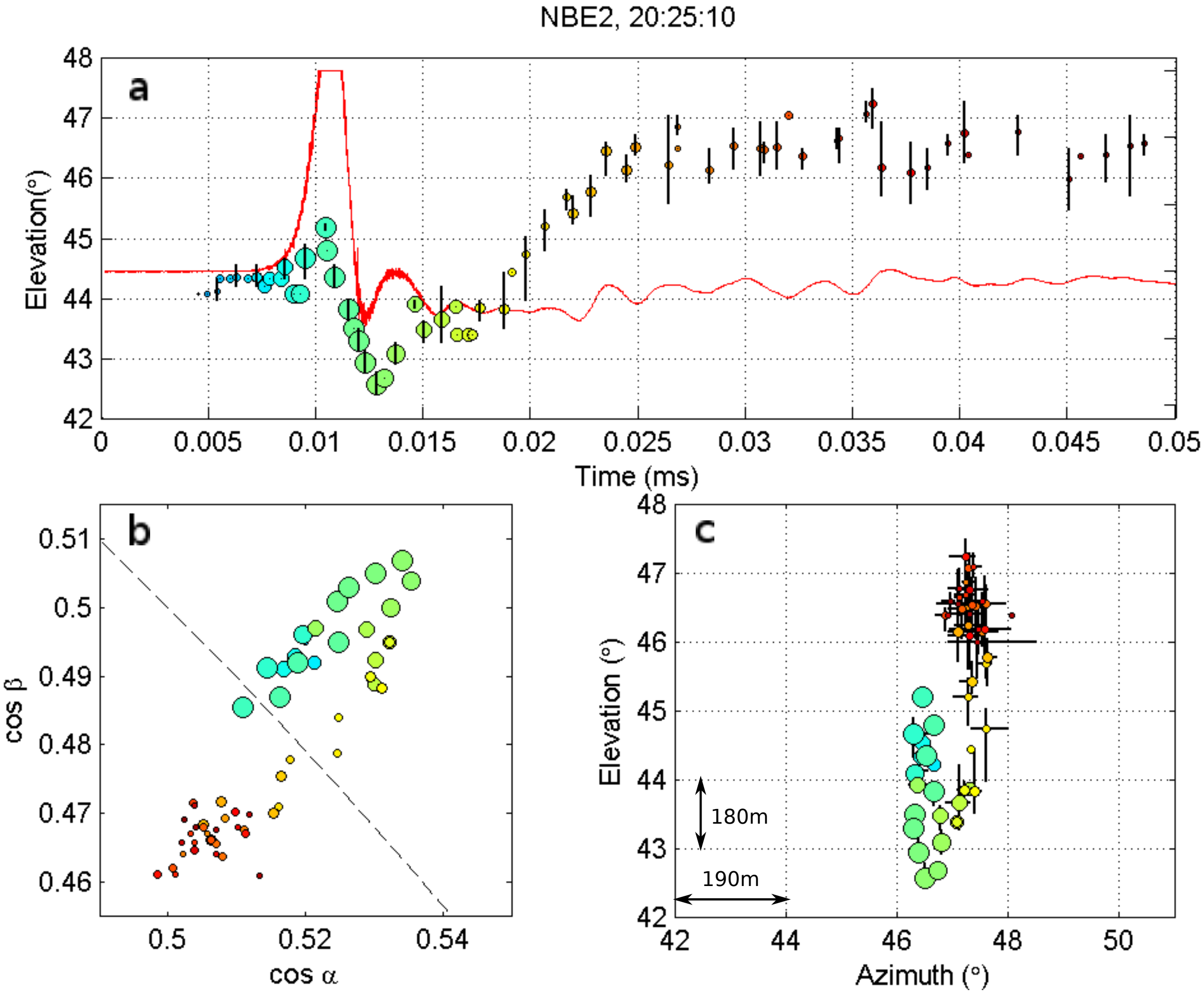

Supplementary Figure 17: Source location uncertainties for NBE2. Same as Supplementary Fig. 16, except for NBE2. Again, the uncertainties of the weak radiation at the beginning of the NBE are small, indicating the initial breakdown was highly localized, and providing a baseline for the uncertainty caused by background noise. The relatively small uncertainties of the sources during the downward NBE indicate that it was primarily a monotonic event, consistent with the NBE's narrower current pulse (Supplementary Fig. 3).

...

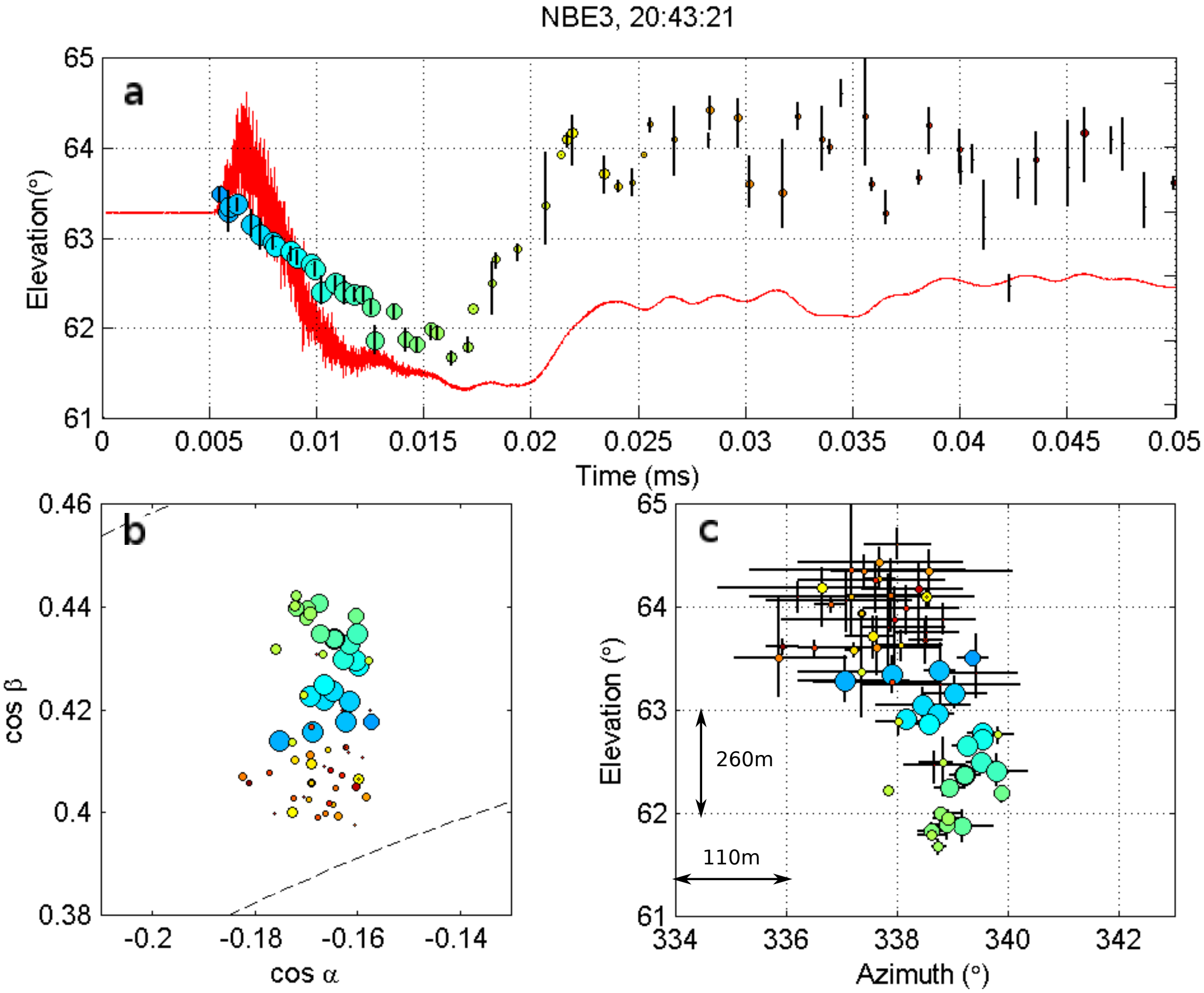

Supplementary Figure 18: Source location uncertainties for NBE3. Same as Fig. 16, except for NBE3. The azimuthal uncertainty of the post-NBE negative breakdown is noticeably larger, indicative of the breakdown having a wider horizontal extent. The azimuthal variability is also significantly larger than for NBEs 1 and 2.

...

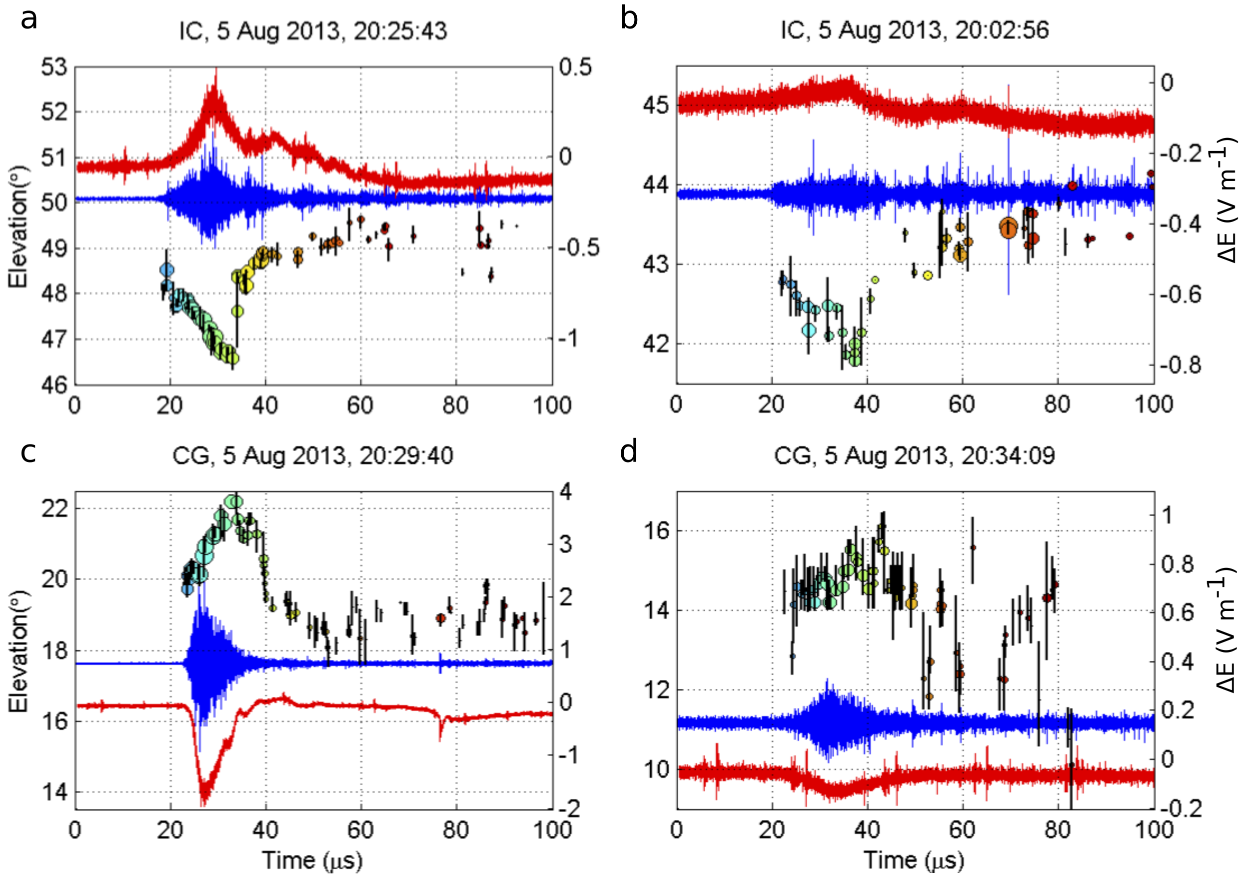

Supplementary Figure 19: Elevation uncertainties for the INTF observations of the intermediate- and weaker-strength NBEs of Fig. 4.

...

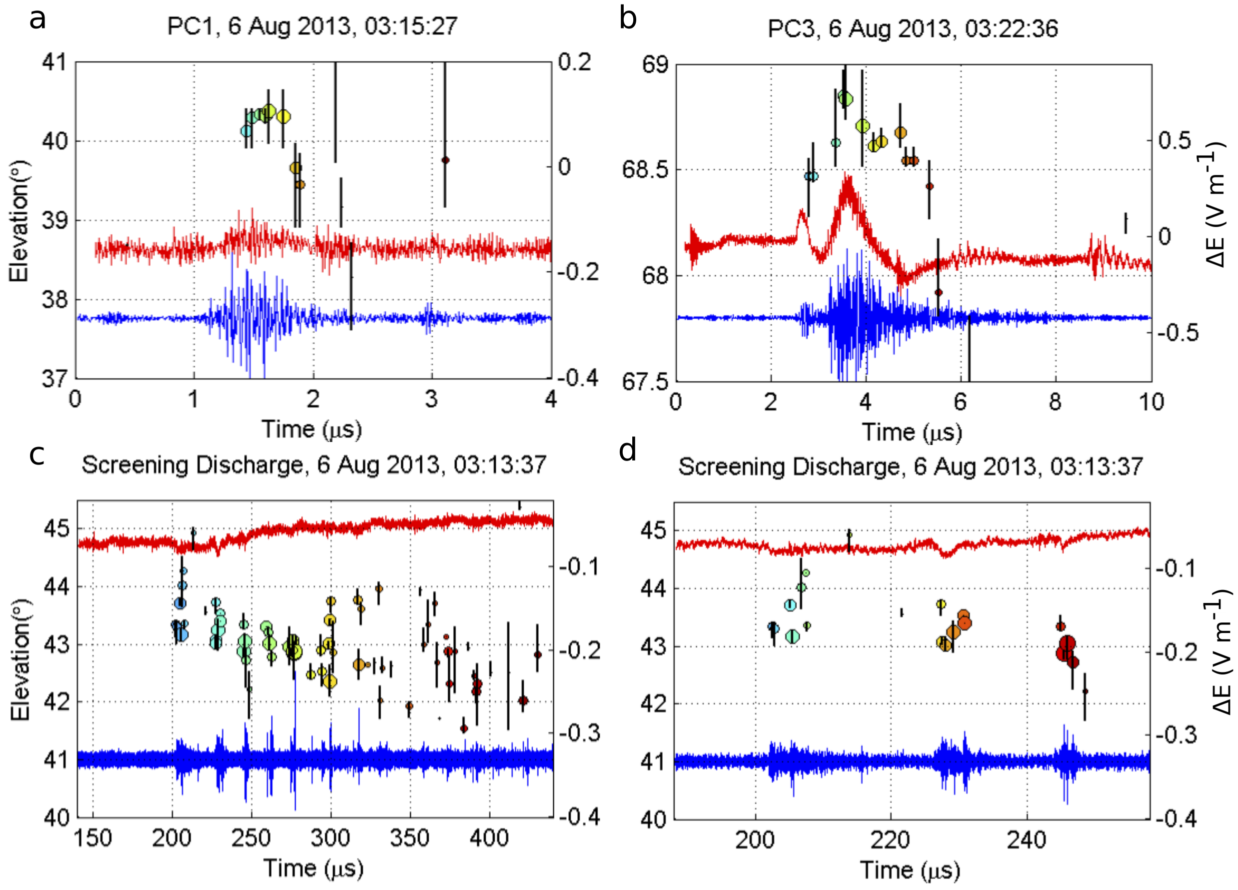

Supplementary Figure 20: **Elevation uncertainties for the precursor and screening discharges of Fig. 6 and Supplementary Fig. 12.**

...

Supplementary Note 1. Positive streamer speeds

Experimental studies of positive streamers in air measure their speeds to be one or two orders of magnitude below the observed speeds of the fast positive breakdown. The highest reported speed is 4×10^6 m s^{-1}, measured by Briels et al.[5] with a 96 kV pulse across a 4 cm point to plane gap at ambient pressure. An approximate average value for the non-uniform electric field would be $\simeq 2.5\times10^6$ m s^{-1}, comparable to the breakdown strength of air (3.2×10^6 V m^{-1}).

Allen and Mikropoulos[6] made detailed observations of the speed of positive streamers in a uniform electric field, and investigated how the propagation speed varied with the ambient field E. The uniform field was provided by a parallel plate arrangement having a 12 cm gap. They expressed the observations in terms of the minimum field E_{st} required to initiate a stable streamer, called the 'stability' field, and the corresponding stability velocity v_{st} of the resulting streamer. An electric field $E_{\mathrm{st}} = 491\cdot\delta$ kV m^{-1} was required to produce a streamer of minimal initial energy, where δ is the fractional air density relative to standard atmospheric conditions. The associated propagation speed was $v_{\mathrm{st}} = 1.25\times10^5$ m s^{-1}. For stronger fields, the streamer speed v_{str} was well-fitted by a cubic power law dependence on E, according to

$$v_{\mathrm{str}} = v_{\mathrm{st}}\left(\frac{E}{E_{\mathrm{st}}}\right)^3 = (1.25\times10^5)\left(\frac{E/\delta}{4.91\times10^5}\right)^3 \text{ m s}^{-1}\,. \tag{1}$$

The measurements were made at ambient pressure with electric fields between 450 and 800 kV m^{-1}. The speed corresponding to 800 kV m^{-1}was between $\simeq 5$ and 10×10^5 m s^{-1}, depending on the amplitude and duration of the pulse used to initiate the streamers from a recessed point in the ground plane (2 and 4 kV, and 135 and 270 ns, respectively).

The question of interest is whether at stronger fields the cubic relation would contine to hold, and whether the propagation speed can reach the observed values obtained for the fast positive breakdown. To answer the question it is instructive to evaluate (1) for the streamer speed that would be predicted if E is assumed to be the breakdown field $E_{\mathrm{k}} = (3.2\times10^6)\cdot\delta$ V m^{-1}. Calling this speed v_{k}, the answer is that the density dependence cancels, giving

$$v_{\mathrm{k}} = (1.25\times10^5)\left(\frac{3.2\times10^6}{4.91\times10^5}\right)^3 = 3.5\times10^7 \text{ m s}^{-1}\,. \tag{2}$$

Remarkably, this is the same as the speeds of NBEs 1 and 3, and is close to the speeds estimated for the lesser-power discharges. It is based entirely on empirical data from laboratory observations made at ground level and over a restricted range of E field values. Agreement with the observations of the present study suggests that (1) is valid for fields up to and possibly beyond breakdown, and that the fast positive breakdown occurs at or near E_{k}, independent of altitude. Taken at face value, E/E_{k} would be slightly above unity when $v_{\mathrm{str}} > v_{\mathrm{k}}$, and slightly less than unity for $v_{\mathrm{str}} < v_{\mathrm{k}}$. In the super-critical case (e.g., for NBE2 or for the screening discharge), the breakdown might be expected take the form of an ionization wave rather than discrete streamers. While this might happen in some circumstances, it should be noted that the value of v_{st} in (1) applies to streamers having minimal initial energy, and that the speeds for a given value of E are increased by a factor of two for initial energies corresponding to relatively small potentials of 2–4 kV (Fig. 10 of Allen and Mikropoulos). The observed speeds would then be attained at fields somewhat below breakdown.

Overall, the picture that emerges is that positive streamers at the beginning of a discharge intensify the electric field up to or near breakdown, and then continue as a result of self-generated field enhancement ahead of the streamer system[7]. Allen and Mikropoulos showed that the streamer speed stabilized within a few cm of being initiated, and noted particularly that speed invariance through the remainder of the gap indicated that branching had negligible effect on the results.

Supplementary Note 2.

Shortly before this paper being accepted, Shi *et al.*[8] reported modelling results that support the findings and inferences of the present study that the fast positive breakdown is streamer-based. Their findings extend results obtained in the earlier study by Liu *et al.*[9] to show that positive streamers a) can be initiated from isolated hydrometeors at reasonable ambient field strengths and storm altitudes, b) exhibit exponential growth up to speeds of $1-5\times10^{7}$ m s^{-1} over short distances (0.3–1.8 MV potential difference), and c) develop oscillatory fluctuations in their wake that would radiate in the high (HF) and very high (VHF) frequency regimes.

Supplementary References

1. Smith, D. A. *et al.* The Los Alamos Sferic Array: A research tool for lightning investigations. *J. Geophys. Res.* **107,** ACL 5-1–5-14 (2002).
2. Stock, M. A. Broadband interferometry of lightning. Ph.D. Dissertation, N.M. Inst. Mining & Tech., Socorro (2014).
3. Rhodes, C. T., Shao, X. M., Krehbiel, P. R., Thomas, R. J., & Hayenga, C. O. Observations of lightning phenomena using radio interferometry. *J. Geophys. Res.* **99,** 13059–13082 (1994).
4. Stock, M. G. *et al.* Continuous broadband digital interferometry of lightning using a generalized cross-correlation algorithm. *J. Geophys. Res. Atmos.* **119,** 3134–3165 (2014).
5. Briels, T. M. P., Kos, J., Winands, G. J. J., van Veldhuizen, E. M. & Ebert, U. Positive and negative streamers in ambient air: measuring diameter, velocity and dissipated energy. *J. Phys. D* **41,** 234004 (2008).
6. Allen, N. L. & Mikropoulos, P. N. Dynamics of streamer propagation in air. *J. Phys. D* **32,** 913919 (1999).
7. Ebert, U. & Sentman, D. D. Streamers, sprites, leaders, lightning: From micro- to macroscales. *J. Phys. D* **41,** 230301 (2008).
8. Shi, F., Liu, N. & Rassoul, H. Dynamics of a relative long positive streamer from an isolated hydrometeor. *AGU Fall Annual Meet.*, Abstract AE14A-01 (2015).
9. Liu, N., Kosar, B., Sadighi, S., Dwyer, J. R. & Rassoul, H. K. Formation of streamer discharges from an isolated ionization column at subbreakdown conditions. *Phys. Rev. Lett.* **109,** 025002 (2012).
